# Humanized V(D)J-rearranging and TdT-expressing mouse vaccine models with physiological HIV-1 broadly neutralizing antibody precursors

**DOI:** 10.1073/pnas.2217883120

**Published:** 2022-12-27

**Authors:** Sai Luo, Changbin Jing, Adam Yongxin Ye, Sven Kratochvil, Christopher A. Cottrell, Ja-Hyun Koo, Aimee Chapdelaine Williams, Lucas Vieira Francisco, Himanshu Batra, Edward Lamperti, Oleksandr Kalyuzhniy, Yuxiang Zhang, Alessandro Barbieri, John P. Manis, Barton F. Haynes, William R. Schief, Facundo D. Batista, Ming Tian, Frederick W. Alt

**Affiliations:** ^a^HHMI, Boston Children's Hospital, Boston, MA 02115; ^b^Program in Cellular and Molecular Medicine, Boston Children’s Hospital, Boston, MA 02115; ^c^Department of Genetics, Harvard Medical School, Boston, MA 02115; ^d^The Ragon Institute of Massachusetts General Hospital, Massachusetts Institute of Technology and Harvard University, Cambridge, MA 02139; ^e^Department of Immunology and Microbiology, The Scripps Research Institute, La Jolla, San Diego, CA 92037; ^f^IAVI Neutralizing Antibody Center, The Scripps Research Institute, La Jolla, San Diego, CA 92037; ^g^Center for HIV/AIDS Vaccine Development, The Scripps Research Institute, La Jolla, San Diego, CA 92037; ^h^Department of Laboratory Medicine, Boston Children’s Hospital, Boston, MA 02115; ^i^Duke Human Vaccine Institute, Duke University School of Medicine, Durham, NC 27710; ^j^Department of Medicine, Duke University School of Medicine, Durham, NC 27710; ^k^Department of Immunology, Duke University School of Medicine, Durham, NC 27710; ^l^Department of Immunology, Harvard Medical School, Boston, MA 02115; ^m^Department of Microbiology, Harvard Medical School, Boston, MA 02115

**Keywords:** VRC01-class broadly neutralizing antibody, humanized mouse model, HIV-1 vaccine, terminal deoxynucleotidyl transferase

## Abstract

Antibody heavy chain (HC) and light chain (LC) variable region exons are assembled by V(D)J recombination. V(D)J junctional regions encode complementarity-determining-region 3 (CDR3), an antigen-contact region immensely diversified through nontemplated nucleotide additions (“N-regions”) by terminal deoxynucleotidyl transferase (TdT). HIV-1 vaccine strategies seek to elicit human HIV-1 broadly neutralizing antibodies (bnAbs), such as the potent CD4-binding site VRC01-class bnAbs. Mice with primary B cells that express receptors (BCRs) representing bnAb precursors are used as vaccination models. VRC01-class bnAbs uniformly use human HC V_H_1-2 and commonly use human LCs Vκ3-20 or Vκ1-33 associated with an exceptionally short 5-amino-acid (5-aa) CDR3. Prior VRC01-class models had nonphysiological precursor levels and/or limited precursor diversity. Here, we describe VRC01-class rearranging mice that generate more physiological primary VRC01-class BCR repertoires via rearrangement of V_H_1-2, as well as Vκ1-33 and/or Vκ3-20 in association with diverse CDR3s. Human-like TdT expression in mouse precursor B cells increased LC CDR3 length and diversity and also promoted the generation of shorter LC CDR3s via N-region suppression of dominant microhomology-mediated Vκ-to-Jκ joins. Priming immunization with eOD-GT8 60mer, which strongly engages VRC01 precursors, induced robust VRC01-class germinal center B cell responses. Vκ3-20-based responses were enhanced by N-region addition, which generates Vκ3-20-to-Jκ junctional sequence combinations that encode VRC01-class 5-aa CDR3s with a critical E residue. VRC01-class-rearranging models should facilitate further evaluation of VRC01-class prime and boost immunogens. These new VRC01-class mouse models establish a prototype for the generation of vaccine-testing mouse models for other HIV-1 bnAb lineages that employ different HC or LC Vs.

## Significance

Mouse models that express human precursors of HIV-1 broadly neutralizing antibodies (bnAbs) are useful for evaluating vaccination strategies for eliciting such bnAbs in humans. Prior models were handicapped by nonphysiological frequency and/or diversity of B lymphocytes that express the bnAb precursors. We describe a new class of mouse models in which the mice express humanized bnAb precursors at a more physiologically relevant level through developmental rearrangement of both antibody heavy- and light-chain gene segments that encode the precursors. The model also incorporated a human enzyme that diversifies the rearranging gene segments and promotes the generation of certain variable region sequences needed for the response. This new class of mouse models should facilitate the preclinical evaluation of candidate HIV-1 vaccination strategies.

Diverse antibody variable region exons are assembled in developing B cells from Immunoglobulin (Ig) heavy chain (HC) V, D, and J gene segments and from Igκ or Igλ light chain (LC) V and J segments (1). In humans, there are 55 germline HC Vs (V_H_s) and 70 Igκ and Igλ LC Vs. Vs encode most of the HC and LC variable region, including the antigen contact CDR1 and CDR2 sequences that vary among different HC and LC Vs. Ig HC V(D)J recombination occurs at the progenitor (Pro) B cell developmental stage in the fetal liver and in the postnatal bone marrow (2, 3). Ig LC V to J recombination takes place in the subsequent precursor (Pre) B cell developmental stage in these same sites (1). T cell receptor (TCR) variable region exon assembly also occurs in the fetal liver and thymus and then in the postnatal thymus (4, 5). Mice also have similar sets of Ig HC and LC and TCR variable region gene segments as those found in humans and, in general, assemble them in the context of similar developmental processes (6, 7).

Primary B cell receptor (BCR) diversity is achieved, in part, by assorting HC and LC Vs along with each of their distinct sets of CDR1 and CDR2 sequences. However, several V(D)J junctional diversification mechanisms play an even greater role in V(D)J diversity generation (8). In this regard, terminal deoxynucleotidyl transferase (TdT), a DNA polymerase that adds nucleotides to 3'DNA ends without a template (9), plays a key role. V(D)J junctional diversity is immensely augmented by TdT-based nontemplated nucleotide additions, referred to as N regions (10), that are added to V(D)J junctions. While N-region addition generates CDR3 length and sequence diversity, it also suppresses recurrent CDR3s resulting from microhomology (MH)-mediated V(D)J joining (10–13). TdT expression is absent during fetal B and T cell development, resulting in less diverse repertoires dominated by variable region exons promoted by recurrent MH-mediated joins (14–21). In contrast, TDT expression diversifies antigen receptor variable region repertoires generated in mouse and human developing B and T cells that develop postnatally, with the notable exception of LC variable region repertoires in mice (10, 22, 23). Thus, while TdT is expressed during LC V(D)J recombination in postnatal human Pre-B cells (24), it is not expressed in postnatal mouse pre-B cells (25, 26), leading to decreased junctional diversity and much more abundant MH-mediated joins in primary mouse LC repertoires compared to those of humans (22, 23). Lack of TdT expression in fetal repertoires also is known to promote recurrent MH-mediated V(D)J junctions, that are not dominant in postnatal repertoires due to TdT expression. Some such recurrent MH-mediated V(D)J joins in fetal T or B cell repertoires generate TCRs or BCRs critical for certain physiological responses (13, 14, 27, 28). However, the potential role of TdT and N regions in promoting specific responses has remained largely unaddressed.

VRC01-class bnAb HCs employ human V_H_1-2, which encodes residues that contact the HIV-1 envelope protein (Env) CD4 binding site (29–37). VRC01-class LC variable regions are known to be encoded by several Vs; but all are associated with an exceptionally short 5 amino acid (5-aa) CDR3, which avoids steric clash with Env and contributes to Env interaction (29–37). As both requirements can be achieved by V(D)J recombination, they are predicted attributes of primary VRC01-class precursor BCRs. However, inferred primary VRC01-class BCRs lack detectable affinity for naive Envs (38–41). In this regard, following BCR antigen-activation, primary B cells are driven into germinal center (GC) reactions where they undergo rounds of variable region exon somatic hyper-mutation (SHM) followed by selection of SHMs that increase BCR antigen-binding affinity. This process ultimately leads to high-affinity antibody production. Correspondingly, a third VRC01-class bnAb attribute is abundant variable region SHMs with only a subset contributing to broad Env-binding and potent VRC01-class bnAb activity (37, 42), consistent with VRC01-class bnAb evolution occurring over long HIV-1 infection times and many SHM/selection cycles.

To elicit VRC01-class broadly neutralizing antibodies (bnAbs), sequential vaccine immunization approaches propose a priming immunogen to drive precursors into GCs followed by boost immunogens designed to lead them through rounds of SHM/affinity maturation. Based on a structurally designed eOD-GT8 immunogen that binds to the inferred VRC01 unmutated common ancestor (UCA) BCR, potential human VRC01-like precursor B cell frequency was estimated to be one in 400,000 or fewer (43, 44). To test the priming and sequential immunogens that could elicit VRC01-class bnAbs in humans, mouse models are needed that reflect as closely as possible the biology of human B cell responses. Early models expressed knock-in V_H_1-2 HCs and, in some, VRC01-class LC Vs, both with mature CDR3s (45–47). These models were nonphysiologic as their BCR repertoire was dominated by a single human HC/LC combination or a single human HC with diverse mouse LCs. Mice with fully human HC and LC gene segment loci assembled by V(D)J recombination were also tested; but precursor frequencies were 150- to 900-fold lower than that of humans (48), likely due to inability to express immense human-like CDR3 repertoires in mice with orders of magnitude fewer B cells. A V_H_1-2-rearranging mouse model generated diverse V_H_1-2 HC CDR3s, but it employed a germline-reverted VRC01 precursor LC with a 5-aa CDR3 from mature VRC01 bnAb (49). While useful for HC maturation studies during sequential immunization, this model was limited by over-abundance of VRC01 lineage LC precursors. More recently, B cells from transgenic VRC01-class UCA or eOD-GT8-binding precursor knock-in mice were adoptively transferred into congenic recipient mice at human-like frequencies (50–53). While this elegant approach has been very useful, it still has certain limitations as it focused only on eOD-GT8-priming and tested just a small subset of potential VRC01 lineage precursors (50–53).

## Results

### Generation of Mice with VRC01-Class-Rearranging Human HC and LC Vs.

**Fig. 1.**
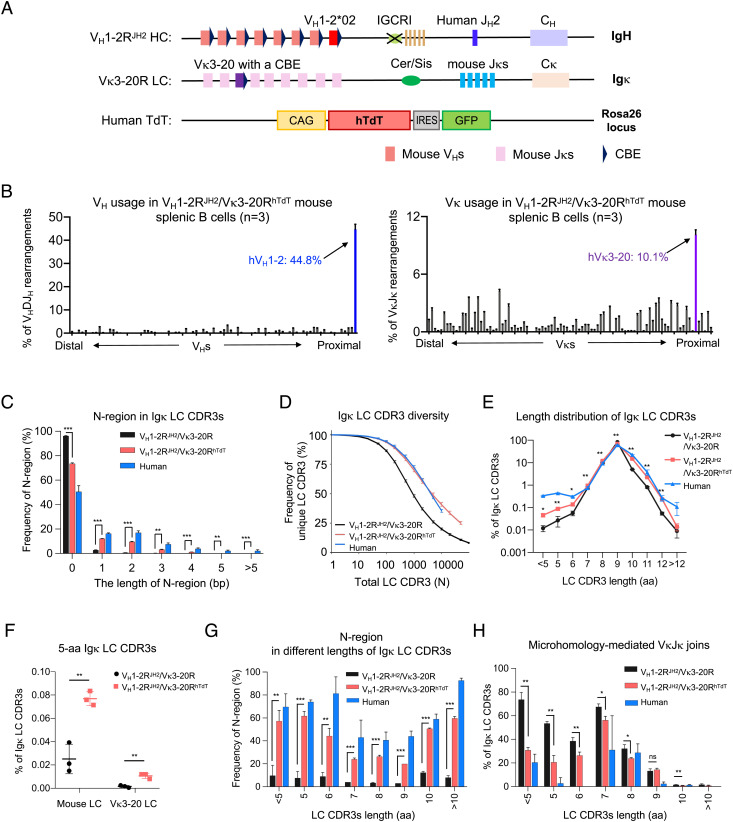
Generation and characterization of the V_H_1-2^JH2^/Vκ3-20^hTdT^-rearranging mouse models. (*A*) Illustration of genetic modifications in the *Igh* and *Igκ* locus of V_H_1-2^JH2^/Vκ3-20-rearranging mouse models. The most D_H_-proximal functional mouse V_H_ (V_H_81X) was replaced with the human V_H_1-2 on an IGCR1-deleted allele. The mouse J_H_s were replaced with the human J_H_2. The *Jκ*-proximal Vκ3-7 was replaced with human Vκ3-20 plus a CBE 50 bp downstream of its RSS. Human TdT gene was knocked into mouse *Rosa* locus. (*B*) HTGTS-rep-seq analysis of V_H_ (*Left*) and Vκ (*Right*) usage in V_H_1-2^JH2^/Vκ3-20^hTdT^-rearranging mouse splenic B cells. The x axis listed all functional V_H_s or Vκs from the distal to the *D-* or *Jκ*-proximal end. The histogram displayed the percent usage of each V_H_ or Vκs among all productive V_H_DJ_H_ or VκJκ rearrangements. The usage of human V_H_1-2 and Vκ3-20 is shown in blue and purple, respectively. (*C*) Length distribution of N regions in VκJκ junctions from human, V_H_1-2R^JH2^/Vκ3-20R mouse, and V_H_1-2R^JH2^/Vκ3-20R^hTdT^ mouse naive B cells. The human naive B cells were isolated from human tonsils using CD19^+^, IgD^+^, CD27^−^, and CD38^−^. (*D*) The diversity of Igκ LC CDR3s in human, V_H_1-2R^JH2^/Vκ3-20R mouse and V_H_1-2R^JH2^/Vκ3-20R^hTdT^ mouse naive B cells. The x axis represents the total Igκ LC CDR3 number (N). The y axis represents the frequency of unique Igκ LC CDR3s among total Igκ LC CDR3s. The differences of CDR3 diversities between V_H_1-2R^JH2^/Vκ3-20R and V_H_1-2R^JH2^/Vκ3-20R^hTdT^ mice are significant when the total CDR3 number is above 50 (P < 0.001 for N ≥ 50). (*E*) Length distribution of Igκ LC CDR3s in human, V_H_1-2R^JH2^/Vκ3-20R mouse, and V_H_1-2R^JH2^/Vκ3-20R^hTdT^ mouse naive B cells. (*F*) The frequency of 5-aa LC CDR3 in V_H_1-2R^JH2^/Vκ3-20R and V_H_1-2R^JH2^/Vκ3-20R^hTdT^ mouse naive B cells. (*G*) Frequency of N regions in different lengths of Igκ LC CDR3s from human and V_H_1-2R^JH2^/Vκ3-20R and V_H_1-2R^JH2^/Vκ3-20R^hTdT^ mouse naive B cells. (*H*) Frequency of MH-mediated VκJκ joins in Igκ LC CDR3s from human and V_H_1-2R^JH2^/Vκ3-20 and V_H_1-2R^JH2^/Vκ3-20R^hTdT^ mouse naive B cells. Data from (*B*, *C*, *E–H*) were mean ± SD of three independent experiments. Statistical comparisons in (*C*, *E–H*) were performed between V_H_1-2R^JH2^/Vκ3-20R and V_H_1-2R^JH2^/Vκ3-20R^hTdT^ mice using a two-tailed unpaired t test. *P < 0.05, **P < 0.01, ***P < 0.001

To address issues of prior models, we developed complete VRC01 mouse models in which individual B cells express one of a multitude of different VRC01 precursors at human-like frequencies, based on enforced rearrangement of both V_H_1-2 and VRC01-class Vκs (Fig. 1*A*). All complete VRC01-class models employ our previously described V_H_1-2-rearranging HC allele in which the most D proximal functional mouse V_H_ (V_H_81X) was replaced with human V_H_1-2 (49, 54). The CTCF-binding site (CBE)-based IGCR1 element in the V_H_ to D interval is also inactivated on this allele, which leads to dominant rearrangement of human V_H_1-2 in an otherwise intact upstream mouse V_H_ locus (55). On this allele, high-level V_H_1-2 utilization in the absence of IGCR1 is mediated by its closely associated downstream CBE element (56). Our new models also use a version of this rearranging HC allele in which the mouse J_H_ segments were replaced with human J_H_2, which can contribute a tryptophan residue (Trp100B) conserved in the HC CDR3 of VRC01-class bnAbs (54). We have retained mouse Ds in the model for reasons we have previously described (57). Briefly, mouse Ds are highly related to certain human Ds and the contribution of D sequences to CDR3s is often obscured by V(D)J recombination-associated junctional diversification mechanisms including nucleotide deletions and N region additions (58). When the replacement allele in our new VRC01-class models is bred to homozygosity, V_H_1-2 rearrangements represent nearly 73.8% of primary V(D)J rearrangements (*SI Appendix*, Fig. S1 *A*, *Upper*). Due to the counter selection of lower frequency upstream mouse V_H_ rearrangements, V_H_1-2 contribution to primary B cell BCR repertoires is reduced to 43% (*SI Appendix*, Fig. S1 *A*, *Bottom*), with immense CDR3 diversity (*SI Appendix*, Fig. S1*B*). Such CDR3 diversity is critical, as V_H_1-2-encoded HC CDR3s were implicated in Env recognition by precursor VRC01-class BCRs and also implicated in the maturation of VRC01-class bnAbs (59, 60).

To generate human Vκ-rearranging LC alleles, we used a strategy similar to that which we used for V_H_1-2, as recently described (57). The CBE-based Cer/Sis element in the Vκ to Jκ interval has been implicated in promoting distal versus proximal Vκ rearrangements (61). To test Cer/Sis functions in more detail, we deleted this element from the wild-type mouse allele and assessed its impact on Vκ rearrangement via our high throughput HTGTS-Rep-seq method (62) (*SI Appendix*, Fig. S2*A*). Homozygous Cer/Sis deletion substantially increased (up to eightfold) the frequency of 7 of the 11 the most *Jκ*-proximal Vκs (*SI Appendix*, Fig. S2*B*). Indeed, these 7 Vκs contributed to the vast majority of the primary BCR repertoire of these mice (*SI Appendix*, Fig. S2*C*), as upstream Vκ rearrangements were essentially abrogated in the absence of Cer/Sis. We note that Vκ3-2 and Vκ3-7 showed the greatest increase in utilization in the absence of Cer/Sis. Our initial plan for our VRC01-rearranging mouse models, analogous to our V_H_1-2 rearranging *Igh* allele (49), was to increase the utilization of human Vκs in the model by introducing them into proximal positions on Cer/Sis-deleted *Igκ* alleles.

We replaced the Vκ3-2 sequence encoding the leader-intron-V sequence with the corresponding sequences of human Vκ1-33 on a wild-type *Igκ* allele (“Vκ1-33-rearranging” allele) and then also deleted Cer/Sis on that allele (“Vκ1-33^CS∆^-rearranging” allele) (57). In these replacement alleles, we maintained the mouse Vκ3-2 sequence upstream of the start codon (ATG) (including the promoter) and the Vκ3-2 downstream sequence starting at the Vκ3-2 recombination signal sequence (RSS). HTGTS-Rep-seq revealed that similarly to Vκ3-2, human Vκ1-33 on homozygous replacement alleles in our VRC01-class models accounted for approximately 2% or 17% of primary Vκ rearrangements in the presence or absence of the Cer/Sis element, respectively (*SI Appendix*, Fig. S3*A*). Vκ1-33 contributed to the splenic BCR repertoire at similar frequencies (approximately 2% and 15%, respectively; *SI Appendix*, Fig. S3*B*). We also generated a “Vκ3-20-rearranging allele” in which mouse proximal Vκ3-7 was replaced with human Vκ3-20 (Fig. 1*A* and *SI Appendix*, Fig. S3 *C* and *D*). When homozygous in mice, the Vκ3-20-rearranging allele contributed about 6% of primary Vκ rearrangements and contributed similar frequencies in splenic BCR repertoires (*SI Appendix*, Fig. S3*E*). We considered these levels sufficiently high to leave Cer/Sis intact for initial experiments.

Based on studies of the *Igh* locus (56), we also inserted CBEs just downstream of the RSSs of the inserted Vκ1-33 and Vκ3-20 gene segments (Fig. 1*A*) (57). However, we found that, compared to the rearrangement frequencies of mouse Vκs they replaced, inserted CBEs had no measurable effect on Vκ1-33 rearrangement either in the presence or absence of Cer/Sis (*SI Appendix*, Figs. S2*C* and S3 *A* and *B*) and only modestly increased Vκ3-20 rearrangement in the presence of Cer/Sis (*SI Appendix*, Figs. S2*C* and S3*E*). The inability of an attached CBE to dominantly increase Vκ1-33 rearrangement in the absence of Cer/Sis suggests that mechanisms underlying CBE-enhanced dominant utilization of proximal V_H_s in the absence of IGCR1 may not similarly operate context of Igκ V(D)J recombination in the absence of Cer/Sis. This notion is consistent with recent findings, published after these models were generated, that indicated that mechanisms that promote long-range V_H_ to DJ_H_ joining are, at least in part, distinct from those that promote long-range Vκ to Jκ joining (63).

We refer to these new VRC01-class mouse models with human V_H_1-2- and Vκ-rearranging ("R") alleles as the V_H_1-2R^JH2^/Vκ1-33R model, the V_H_1-2R^JH2^/Vκ1-33R^CS∆^ model (^"CS∆"^ indicates Cer/Sis deletion), and the V_H_1-2R^JH2^/Vκ3-20R model. Based on fluorescence-activated cell sorting (FACS) analyses of cell surface markers, splenic B and T cell populations in all three models were comparable to those of wild-type mice (*SI Appendix*, Fig. S3*F*). During our studies of the V_H_1-2R^JH2^/Vκ3-20R model, we discovered that the inserted Vκ3-20 sequence had acquired a single in-frame point mutation in CDR1 that changes an S to I residue (AGT to ATT) (*SI Appendix*, Fig. S4). We then corrected this mutation in the Vκ3-20 allele, introduced it into all mouse models described, and repeated all experiments originally performed with the mutated allele with mouse models harboring the corrected allele. Based on FACS analyses of cell surface markers, splenic B and T cell populations in the Vκ3-20 corrected model were also comparable to those of wild-type mice and those of the mouse models harboring mutated Vκ3-20 sequence (*SI Appendix*, Fig. S3*F*). Indeed, in all experiments described below, mouse models harboring the mutated and corrected Vκ3-20 sequence gave very similar results with respect to Vκ3-20-based VRC01-class responses, which, for comparison, are included in all immunization experiments and related figures described below.

### Enforced Human TdT Expression Diversifies LC Repertoires.

VRC01-class bnAb LCs commonly have a LC 5-aa CDR3 with a relatively conserved QQYEF amino acid sequence (32, 64). However, as compared to the frequency of LC 5-aa CDR3s in human BCR repertoires, our initial VRC01-class mouse models had 20- to 50-fold lower frequencies of LC 5-aa CDR3s (0.02%) in their mouse Vκ and human Vκ1-33 or Vκ3-20 LC BCR repertoires (*SI Appendix*, Fig. S5*A*) (48, 64). In this regard, approximately 80% of human LC 5-aa-CDR3s are encoded by sequences with hTdT-generated N regions (*SI Appendix*, Fig. S5*B*). Thus, to enforce more human-like TdT expression in mouse bone marrow precursor B cells which normally lack TdT expression, we targeted human hTdT into the *Rosa* locus of ES cells containing the Vκ3-20R allele (Fig. 1*A* and *SI Appendix*, Fig. S5 *C* and *D*); as *Rosa* and *Igκ* both lie on chromosome 6, these two modifications are linked in subsequent crosses. Mice harboring the resulting Vκ3-20R^hTdT^-modified chromosome were bred to homozygosity with the V_H_1-2R^JH2^ allele to create V_H_1-2R^JH2^/Vκ3-20R^hTdT^ mice. The V_H_1-2R^JH2^/Vκ3-20R^hTdT^ mice indeed now expressed human TdT in their progenitor and precursor B cell population (*SI Appendix*, Fig. S5 *E* and *F*). HTGTS-Rep-seq revealed that enforced TdT expression modestly increased Vκ3-20 expression and had little impact on the utilization of V_H_1-2 in splenic B cell populations (Fig. 1*B* and *SI Appendix*, Fig. S5*G*).

As compared to splenic B cells of V_H_1-2R^JH2^/Vκ3-20R mice, those of V_H_1-2R^JH2^/Vκ3-20R^hTdT^ mice had markedly increased frequencies of N regions in both mouse Vκ to Jκ junctions and human Vκ3-20 to Jκ junctions (Fig. 1*C*), and, correspondingly, much more diverse CDR3s (Fig. 1*D*). Notably, while enforced N region addition increased the proportion of longer LC CDR3s (>9-aa), it also increased, up to fivefold, the proportion of short mouse and Vκ3-20 LC CDR3s (<7-aa), including 5-aa CDR3s (Fig. 1 *E* and *F*). Correspondingly, the proportion of N-regions in short LC CDR3s was significantly increased (Fig. 1*G*) and the proportion of MH-mediated short Vκ to Jκ joins (<7-aa) was significantly reduced in splenic B cells of V_H_1-2R^JH2^/Vκ3-20R^hTdT^ mice as compared to those of V_H_1-2R^JH2^/Vκ3-20R mice (Fig. 1*H*). In addition, we compared the LC CDR3s in splenic B cells of V_H_1-2R^JH2^/Vκ3-20R and V_H_1-2R^JH2^/Vκ3-20R^hTdT^ mice to those in human tonsil naive B cells and found that enforced TdT expression in V_H_1-2R^JH2^/Vκ3-20R^hTdT^ mice yielded more human-like CDR3s (Fig. 1 *C*–*E*, *G*, and *H*). As endogenous mouse TdT expression is already robust in V_H_1-2R^JH2^/Vκ3-20R progenitor-stage B cells that undergo HC locus V(D)J recombination, human TdT expression had no obvious effect on HC CDR3 length and diversity in V_H_1-2R^JH2^/Vκ3-20R^hTdT^ mice (*SI Appendix*, Fig. S5 *H* and *I*).

We similarly introduced hTdT into the *Rosa* locus of V_H_1-2R^JH2^/Vκ1-33R^CS∆^ mice and generated V_H_1-2R^JH2^/Vκ1-33R^CS∆/hTdT^ mice. Analyses of splenic B cells from these two models revealed little effect of enforced hTdT expression on overall Vκ1-33 utilization and V_H_1-2 utilization in splenic B cell populations (*SI Appendix*, Fig. S6*A*). However, as in the V_H_1-2R^JH2^/Vκ3-20R^hTdT^ model, Vκ1-33 LC CDR3 diversity and the frequency of Vκ1-33 5-aa CDR3s were significantly increased after hTdT expression (*SI Appendix*, Fig. S6 *B* and *C* ).

### Human TdT Enhanced VRC01-Class GC Responses Induced by eOD-GT8.

**Fig. 2.**
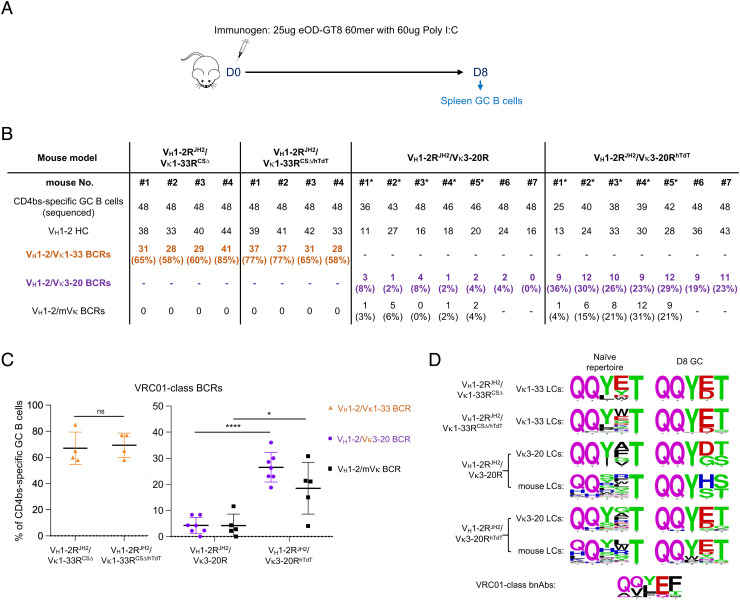
Enforced hTdT expression enhances the VRC01-class GC responses induced by eOD-GT8 60mer. (*A*) Immunization scheme (see text for details). (*B*) Summary of all VRC01-class BCR sequence information obtained from eOD-GT8 immunization in V_H_1-2R^JH2^/Vκ1-33R^CS∆^, V_H_1-2R^JH2^/Vκ1-33R^CS∆/hTdT^, V_H_1-2R^JH2^/Vκ3-20R, and V_H_1-2R^JH2^/Vκ3-20R^hTdT^ mice. VRC01-class BCRs were defined by V_H_1-2 HCs pairing with Vκ1-33/Vκ3-20/mouse LCs with 5-aa CDR3s. Statistical analyses are shown in (*C*). * indicates the mouse harboring a mutated Vκ3-20 allele. (*C*) The frequency of VRC01-class BCRs expressed in CD4bs-specific GC B cells from V_H_1-2R^JH2^/Vκ1-33R^CS∆^, V_H_1-2R^JH2^/Vκ1-33R^CS∆/hTdT^, V_H_1-2R^JH2^/Vκ3-20R, and V_H_1-2R^JH2^/Vκ3-20R^hTdT^ mice. Each point represents one mouse. *P* values were calculated by unpaired, two-tail *t* test. *P < 0.05, **P < 0.01, ***P < 0.001**,** ****P < 0.0001. (*D*) 5-aa LC CDR3 sequence logos for Vκ1-33, Vκ3-20 and mouse LCs in naive BCRs (*Left* column) and eOD-GT8 60mer-induced VRC01-class BCRs at day 8 postimmunization (*Right* column). The sequences of 5-aa LC CDR3s in naive B cells were derived from HTGTS-rep-seq data shown in Fig. 1*B* and *SI Appendix*, Figs. S3 *B* and *E* and S6*A*. The sequences of 5-aa LC CDRs in eOD-GT8 60mer-induced VRC01-class BCRs were recovered from V_H_1-2R^JH2^/Vκ1-33R^CS∆^, V_H_1-2R^JH2^/Vκ1-33R^CS∆/hTdT^, V_H_1-2R^JH2^/Vκ3-20R, and V_H_1-2R^JH2^/Vκ3-20R^hTdT^ mice shown in (*B*). For comparison, the 5-aa LC CDR3 sequences for VRC01-class bnAbs are shown in *Bottom*.

To test if the human TdT expression affects the VRC01-class GC response, we immunized V_H_1-2R^JH2^/Vκ3-20R, V_H_1-2R^JH2^/Vκ1-33R^CS∆^, V_H_1-2R^JH2^/Vκ3-20R^hTdT^, and V_H_1-2R^JH2^/Vκ1-33R^CS∆/hTdT^ mice with eOD-GT8 60mer and poly I:C adjuvant (Fig. 2*A*). All mice developed CD4-binding site (CD4bs)-specific GC responses by day 8 post-immunization, as demonstrated by the presence of GC B cells that bound eOD-GT8 but not ∆eOD-GT8 (which is a VRC01-class epitope knockout variant) (*SI Appendix*, Fig. S7 *A*–*C*). We flow-sorted eOD-GT-specific GC B cells and sequenced their BCRs (Fig. 2*B*). We refer to B cells with VRC01-class BCRs (V_H_1-2 HCs and LCs with 5-aa CDR3s) as VRC01/Vκ1-33, VRC01/Vκ3-20, and VRC01/mVκ B cells, according to the LC they express. At day 8, VRC01/Vκ3-20 and VRC01/mVκ represented 5% and 4%, respectively, of CD4bs-specific GC B cells in V_H_1-2R^JH2^/Vκ3-20R mice and 28% and 20%, respectively, in V_H_1-2R^JH2^/Vκ3-20R^hTdT^ mice (Fig. 2 *B* and *C*). Thus, enforced TdT expression in V_H_1-2R^JH2^/Vκ3-20R line increases the frequency of VRC01-class GC B cells by approximately fivefold. At day 8, VRC01/Vκ1-33 GC B cells represented up to 70% of CD4bs-specific GC B cells in both V_H_1-2R^JH2^/Vκ1-33R^CS∆^ and V_H_1-2R^JH2^/Vκ1-33R^CS∆/hTdT^ mice but no mouse VRC01/mVκs B cells were observed (Fig. 2 *B* and *C*). The lack of mouse VRC01/mVκs B cells in the GCs of immunized V_H_1-2R^JH2^/Vκ1-33R^CS∆^ and V_H_1-2R^JH2^/Vκ1-33R^CS∆/hTdT^ mice probably results from domination of the response by VRC01/Vκ1-33 B cells.

On day 8 post-immunization, the Glu96 (E), a conserved residue in 5-aa LC CDR3s of VRC01-class bnAbs, was dominantly selected by eOD-GT8 in VRC01-class 5-aa LC CDR3s from V_H_1-2R^JH2^/Vκ1-33R^CS∆^, V_H_1-2R^JH2^/Vκ1-33R^CS∆/hTdT^, and V_H_1-2R^JH2^/Vκ3-20R^hTdT^ mice but not from V_H_1-2R^JH2^/Vκ3-20R mice (Fig. 2*D* and *SI Appendix*, Fig. S7*D*). This finding indicated that Vκ to Jκ joining events involving Vκ3-20 or mouse Vκs in the Vκ3-20 mice require N regions added by hTdT to generate the critical E residue in the VRC01-class 5-aa CDR3. Examination of Vκ3-20 and mouse Vκ sequences proved that this is the case (*SI Appendix*, Fig. S7*E*). On the other hand, examination of the Vκ1-33 sequences confirms that they can directly form the E residue in the VRC01-class 5-aa CDR3 when joined to mouse Jκ1 and human Jκ1 in the absence of hTdT activity (*SI Appendix*, Fig. S7*E*). Lack of this E residue in 5-aa mouse LC CDR3s in primary GCs that arose after a single eOD-GT8 immunization was also noted in prior studies (46, 49, 65, 66). Thus, hTdT expression substantially enhanced the VRC01/Vκ3-20 and VRC01/mVκ GC response to eOD-GT8 immunization by generating Vκ3-20-based VRC01-class 5-aa CDR3s that, as a result of N-region addition, have the capacity to encode the critical CDR3 E residue.

### Generation of V_H_1-2^JH2^/Vκ1-33/Vκ3-20^hTdT^-Rearranging Mice.

**Fig. 3.**
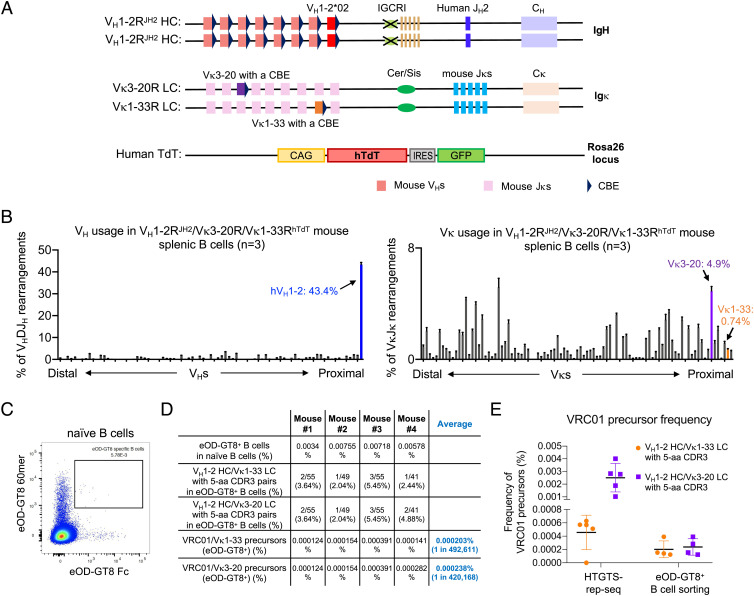
Generation and characterization of the V_H_1-2^JH2^/Vκ1-33/Vκ3-20^hTdT^-rearranging mouse models. (*A*) Illustration of genetic modifications in the *Igh* and *Igκ* locus of V_H_1-2^JH2^/Vκ1-33/Vκ3-20^hTdT^-rearranging mouse models. The *Jκ*-proximal Vκ3-2 was replaced with human Vκ1-33 plus a CBE 50bp downstream of its RSS. (*B*) HTGTS-rep-seq analysis of V_H_ (*Left*) and Vκ (*Right*) usage in V_H_1-2^JH2^/Vκ1-33/Vκ3-20^hTdT^-rearranging mouse splenic B cells. The x axis listed all functional V_H_s or Vκs from the distal to the *D-* or *Jκ*-proximal end. The histogram displayed the percent usage of each V_H_ or Vκs among all productive V_H_DJ_H_ or VκJκ rearrangements. The usage of human V_H_1-2, Vκ1-33, and Vκ3-20 were shown in blue, orange, and purple, respectively. Data from (*A*) and (*B*) were mean ± SD of five libraries from different mice. (*C*) FACS analyses of eOD-GT8-specific naive B cells in V_H_1-2^JH2^/Vκ1-33/Vκ3-20^hTdT^-rearranging mouse. The boxed eOD-GT8-specific naive B cells were sorted for single-cell sequencing. (*D*) Summary of VRC01 precursor sequence information obtained from naive B cell repertoire. The eOD-GT8-specific B cells were defined by eOD-GT8 60mer^+^ and eOD-GT8 Fc^+^. The final frequency of VRC01 precursors in V_H_1-2^JH2^/Vκ1-33/Vκ3-20^hTdT^-rearranging mouse models is 1 in 226,757, approximately. (*E*) Frequency of VRC01 precursors in V_H_1-2^JH2^/Vκ1-33/Vκ3-20^hTdT^-rearranging mice measured by HTGTS-rep-seq or eOD-GT8-specific B cell sorting. The VRC01 precursors were defined by V_H_1-2 HCs pairing with Vκ1-33 and Vκ3-20 LCs with 5-aa CDR3s.

We bred the V_H_1-2R^JH2^/Vκ1-33R^CS∆/hTdT^ and V_H_1-2R^JH2^/Vκ3-20R^hTdT^ mouse lines together to make an even more human-like model that rearranges both VRC01-class Vκs. In this new V_H_1-2R^JH2^/Vκ1-33R^CS∆/hTdT^/Vκ3-20R^hTdT^ mouse model, Vκ1-33 and Vκ3-20 LCs were expressed in 7.8% and 3.4% of splenic B cells, respectively (*SI Appendix*, Fig. S8*A*). However, on day 8 postimmunization with eOD-GT8 60mer, VRC01/Vκ3-20 GC B cells were outcompeted by VRC01/Vκ1-33 GC B cells and were hardly represented in GCs, suggesting the frequency or affinity of responding VRC01/Vκ1-33 precursors was much higher than that of VRC01/Vκ3-20 precursors in this model (*SI Appendix*, Fig. S8*B*). Thus, we further generated the V_H_1-2R^JH2^/Vκ1-33R/Vκ3-20R^hTdT^ model, in which Cer/Sis is still present on the Vκ1-33 allele, leading to a reduction in Vκ1-33 LC-expressing splenic B cell frequency to 0.74% (Fig. 3 *A* and *B*). Indeed, the relative frequency of Vκ1-33 versus Vκ3-20 expressing splenic B cells in the V_H_1-2R^JH2^/Vκ1-33R/Vκ3-20R^hTdT^ model are more comparable to that of humans (67). To assess the frequency of VRC01-precursors, we sorted eOD-GT8-specific naive B cells and identified their BCR sequences (Fig. 3*C* and *SI Appendix*, Fig. S8*C*). The frequency of eOD-GT8-specific VRC01 precursors using Vκ1-33 or Vκ3-20 LCs in this mouse model was approximately 1 in 230,000 (VRC01/Vκ1-33: 1 in 500,000; VRC01/Vκ3-20: 1 in 420,000) (Fig. 3*D*), which is comparable to approximately 1 in 400,000 frequency of eOD-GT8-specific VRC01 precursors measured in humans (44). We also estimated the VRC01-precursor based on HTGTS-Rep-seq data by multiplying the frequency of V_H_1-2 HCs by the frequency of Vκ1-33 and Vκ3-20 LCs with 5-aa CDR3s (Fig. 3*E*). The results suggest that only a small proportion of B cells expressing V_H_1-2 HCs and Vκ3-20 LCs with 5-aa CDR3s bound to eOD-GT8.

### VRC01-Class B Cells Develop SHM and Affinity Maturation in GCs Induced by eOD-GT8 60mer.

**Fig. 4.**
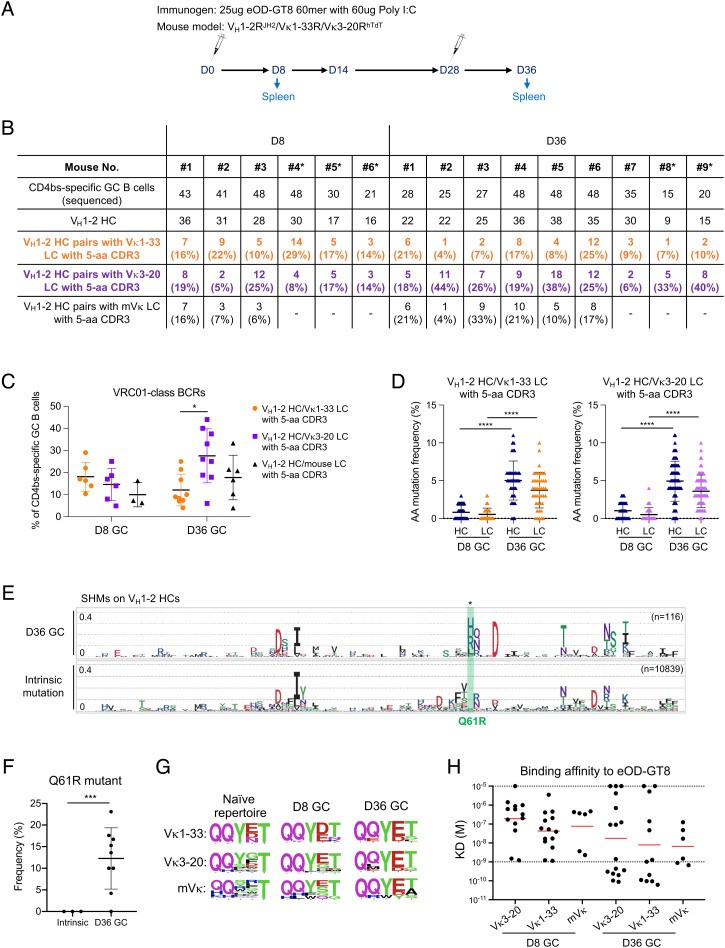
Strong VRC01-class GC responses induced by eOD-GT8 60mer in V_H_1-2^JH2^/Vκ1-33/Vκ3-20^hTdT^-rearranging mouse models. (*A*) Immunization scheme (see text for details). (*B*) Summary of all VRC01-class BCR sequence information obtained from eOD-GT8 60mer immunization at day 8 and day 36. VRC01-class BCRs were defined by V_H_1-2 HCs pairing with Vκ1-33/Vκ3-20/mouse LCs with 5-aa CDR3s. Statistical analyses are shown in (*C*). * indicates the mouse harboring a mutated Vκ3-20 allele. (*C*) The frequency of VRC01-class BCRs expressed in CD4bs-specific GC B cells from day 8 and day 36 GCs of V_H_1-2R^JH2^/Vκ1-33R/Vκ3-20R^hTdT^ mice. (*D*) Amino acid mutation frequency in VRC01-class antibodies cloned from day 8 and day 36 GCs of V_H_1-2R^JH2^/Vκ1-33R/Vκ3-20R^hTdT^ mice. Each dot represents one HC or one LC. The median with interquartile range is plotted. (*E*) Mutation frequency of each amino acid on germline-encoded V_H_1-2 region of VRC01-class antibodies cloned from day 36 GCs shown in sequence logo profiles. For reference, the intrinsic mutation patterns from nonproductive rearrangements derived from Rep-SHM-seq data are represented on the bottom (see *Method* for details). The distance between dotted horizontal lines representing 0.1 (10%). The Q61R mutant is labeled in green. (*F*) Frequency of Q61R mutant on day 36 V_H_1-2 HC compared to that in intrinsic mutation patterns. (*G*) 5-aa LC CDR3 sequences in naive repertoire and VRC01-class antibodies cloned from day 8 and day 36 GCs induced by eOD-GT8 60mer. 5-aa LC CDR3 sequence logos for Vκ1-33, Vκ3-20, and mouse LCs in naive BCRs (*Left* column), 8-d GCs (*Middle* column), and 36-d GCs (*Right* column) induced by eOD-GT8 60mer. (*H*) eOD-GT8 dissociation constants measured by surface plasmon resonance (SPR) for eOD-GT8 60mer elicited VRC01-class antibodies (see *Method* and *SI Appendix*, Tables for details). Data are shown for VRC01-class antibodies from 8-d and 36-d GCs. Bars represent geometric mean (red). Statistical comparisons in (*C*), (*D*), and (*F*) were performed using a two-tailed unpaired *t* test. *P <0.05, **P <0.01, ***P <0.001, ****P <0.0001

To test if V_H_1-2R^JH2^/Vκ1-33R/Vκ3-20R^hTdT^ mice respond to the VRC01-class prime immunogens and support affinity maturation of VRC01-class GC B cells at sufficient levels to support future prime-boost studies, we immunized them with eOD-GT8 60mer and then boosted them with eOD-GT8 60mer at day 28 (Fig. 4*A*). VRC01/Vκ1-33, VRC01/Vκ3-20, and VRC01/mVκ B cells were highly enriched in CD4bs-specific GC B cells at both 8 d and 36 d postimmunization (Fig. 4*B* and *SI Appendix*, Fig. S9 *A*–*C*). Evaluation of GC responses at day 8 and day 36 revealed that the frequencies of VRC01/Vκ3-20 GC B cells and VRC01/Vκ1-33 GC B cells were comparable at day 8, but the frequencies of VRC01/Vκ3-20 GC B cells was higher than that of VRC01/Vκ1-33 GC B cells at day 36 (Fig. 4*C*). Sequencing analyses of VRC01-class antibodies cloned from both day 8 and day 36 GCs revealed extensive SHM, with a maximum of 17 aa mutations and a median of 9 aa mutations at day 36 (Fig. 4*D* and *SI Appendix*, Fig. S9 *D* and *E*), and wide ranges of HC CDR3 length (*SI Appendix*, Fig. S9*F*). To further analyze VRC01-class GC B cell sequence mutations, we compared them to intrinsic mutation patterns generated from nonproductive rearrangements of GC B cells without affinity selection (Fig. 4*E* and *SI Appendix*, Fig. S9 *G*–*I*) (see *Method*) (68). The Q61R mutant on the V_H_1-2 HC reported for VRC01-class bnAbs was significantly enriched in day 36 VRC01-class antibodies (Fig. 4*F*) (42). The Glu96 (E) residues in LC CDR3s were dominant in all types of day 36 VRC01-class antibodies (Fig. 4*G*). We expressed several VRC01-class antibodies with different LCs cloned from day 8 and day 36 GCs. Antibodies from day 8 GCs showed a range of binding affinities, with a median of 100 nM K_D_, to eOD-GT8 (Fig. 4*H*). For the antibodies from day 36 GCs, about 50% showed much higher binding activities, below 1 nM K_D_, representing an average affinity improvement of 100-fold (Fig. 4*H* and *SI Appendix*, Table S1). Altogether, our findings strongly indicate that the V_H_1-2R^JH2^/Vκ1-33R/Vκ3-20R^hTdT^ VRC01-class and related models will facilitate testing prime-boost immunization strategies aimed to advance eOD-GT8-primed vaccination studies to be used in human clinical trials.

## Discussion

Many prior mouse models employed to test vaccine strategies designed to elicit VRC01-class HIV-1 bnAbs had exceedingly high or extremely low levels of VRC01-class precursor B cells (45–49). Other approaches to generate more physiological levels of VRC01 precursors in mouse models were limited by being designed to test only the eOD-GT8 priming immunogen in the context of very limited precursor diversity (50, 51). We have now described more physiologically relevant VRC01-class V(D)J-rearranging mouse models for testing priming and boosting strategies designed to elicit VRC01-class bnAbs. These new VRC01-class rearranging mouse models rearrange both human VRC01-class V_H_1-2 and Vκ3-20 and/or Vκ1-33 variable region gene segments, along with mouse V_H_s and Vκs during normal B cell development. The various mouse lines generated to make the VRC01-class rearranging models described here employ several different genetic strategies that should allow titration of the expression level of diverse Vκ3-20- and/or Vκ1-33-based variable region exons to establish mouse models that generate VRC01 precursor B cells over a wide range of levels (*SI Appendix*, Table S2). Of these models, the V_H_1-2R^JH2^/Vκ1-33R/Vκ3-20R^hTdT^ model, described in depth in this report, generates a highly diverse set of potential VRC01-class precursors in mouse repertoires at similar relative levels to those found in human B cell repertories. Importantly, the potential VRC01-class precursors with highly diverse CDR3s generated in the VRC01-class rearranging models should not be biased with respect to evaluating the efficacy of any particular VRC01-class priming immunogen (*SI Appendix*, Fig. S9*F*).

In this initial study, we have tested the eOD-GT8 priming immunogen in several VRC01 class rearranging models, including the most human-like V_H_1-2R^JH2^/Vκ1-33R/Vκ3-20R^hTdT^ model and found robust engagement of VRC01-class precursors into GCs where they generated equally robust eOD-GT8-specific responses. Other types of priming immunogens that may not be as robust in engaging VRC01-class precursors as eOD-GT8, such as 426c-degly3 Ferritin (40, 47) or GT1 trimer (69), should also be able to be readily evaluated in our new models. Conceivably, studies of some VRC01-class immunogens that have lower affinity for precursors may benefit initially through the use of VRC01-class models that express higher levels of VRC01-class precursors (*SI Appendix*, Table S2). Also, as individual VRC01-class precursor B cells in these new VRC01-class rearranging models express one of a multitude of different variations of the potential VRC01 precursors, they may, in theory, be useful for identifying new pathways that could lead to the generation of potent VRC01-class bnAbs. For any tested priming immunogen that generates a response, our new models could also be used to test sequential boost immunogens designed to lead them through rounds of SHM/affinity maturation that drive responses toward the generation of VRC01-class bnAbs, as described for less diverse earlier versions of these models (49, 70).

A key feature of our new models is their ectopic TdT expression that forces their mouse Pre-B cells to further diversify their mouse and human LC variable region repertoires and make them more human-like, both with respect to contributing N-region diversity and by dampening recurrent MH-mediated join levels in their postnatal LC repertoires. As mentioned, the absence of TdT in fetal repertoires promotes recurrent MH-mediated junctions that lead to the generation of particular Ig or TCR variable region exon sequences (14–21). For example, generation of recurrent “canonical” joins in fetal repertoires in the absence of TdT and N region additions underlies the generation of canonical junctions encoding recurrent γ/δ TCRs expressed on “innate-like” intraepithelial γ/δ T cells that persist into adulthood in both mice and humans (71, 72). Notably, enforced TdT expression during fetal lymphocyte development dampens some such responses (13, 28). In this study, we found that enforced TDT expression in mouse Pre-B cells increased the frequency of short 5-aa CDR3 sequences, such as those used in a VRC01-class response, and promoted a specific Vκ3-20-based eOD-GT8 primary response by generating N sequences that contribute to encoding a critical VRC01 class 5-aa CDR3 residue. Analyses of human Vκ3-20-based VRC01-class sequences indicate that this mechanism also operates in humans (e.g., *SI Appendix*, Fig. S7*E*). By extension, it is likely that postnatal TdT expression in mouse developing B cells will similarly contribute to other responses.

The strategies we employed for constructing the VRC01 rearranging mouse model can be generally adopted for generating mouse models for other classes of anti-HIV-1 bnAbs. In this regard, CDR3 diversification, including engineering the models to make very long human CDR3s, will be especially relevant for testing immunogens for bnAbs that rely heavily on CDR3 to contact Env epitopes, such as those of the V2 apex, V3 glycan, and MPER classes (73). The limitations with previously employed strategies to generate mouse models to test VRC01-class immunization strategies outlined above also will apply to mouse models designed to test immunogens in the context of these other bnAb lineages. Beyond this, all straight precursor variable region knock-in strategies are limited by difficulty in accurately inferring the CDR3 of the UCA sequence of precursors, which may include contributions from both nontemplated nucleotides and somatic hypermutations (74). Indeed, due to the enormous CDR3 diversity in human antibody repertoires, a specific bnAb precursor may not be present in all individuals. To work at a population level, a vaccine should stimulate B cells expressing a range of related precursors. Mouse models expressing a unique bnAb precursor cannot assess this critical parameter. Also, the expression of certain bnAb precursor HCs or LCs can interfere with B cell development, leading to B cell deletion in bone marrow and/or anergy in peripheral lymphoid tissues (73, 75–77). The prototype VRC01-class rearranging mouse model we have described here addresses these potential issues in the VRC01 lineage. Thus, V(D)J recombination generates human VRC01-class precursors that express highly diverse CDR3s, many of which may be compatible with bnAb development. This type of mouse HIV-1 vaccine model does not depend on UCA inference. Additionally, the CDR3 diversity in the model facilitates the assessment of the ability of immunogens to tolerate CDR3 flexibility and mobilize related precursors for bnAb development. Finally, by generating diverse human primary BCR repertoires, rearranging mouse models can provide precursors that support normal B cell development and, correspondingly, generate B cells responsive to immunization.

## Materials and Methods

### VRC01-Rearranging Mouse Model and Embryonic Stem Cells.

The genetic modifications in the *Igκ* locus were introduced into previously generated V_H_1-2 ES cells (129/Sv and C57BL/6 F1 hybrid background), using targeting strategies described previously (49). The mouse Vκ3-7 segment was replaced with human Vκ3-20 segment with an attached CBE (atccaggaccagcagggggcgcggagagcacaca) inserted 50 bp downstream of human Vκ3-20 segment. The replacement was mediated by homologous recombination using a PGKneolox2DTA.2 (Addgene #13449) construct and one guide RNA that targeted the mouse Vκ3-7 segment. The human TdT cDNA was cloned into the CTV (Addgene #15912) construct in which the TdT expression was driven by the CAG promotor and followed by an EGFP expression that is mediated by an internal ribosome entry site (IRES) (78). The TdT expression cassette was inserted into the first intron of mouse Rosa26 gene which is on the same chromosome 6 with *Igκ* locus by homologous recombination. The sequence of guide RNA used for targeting was listed in *SI Appendix*, Table S3. The ESCs were grown on a monolayer of mitotically inactivated mouse embryonic fibroblasts (iMEF) in DMEM medium supplemented with 15% bovine serum, 20 mM HEPES, 1× MEM nonessential amino acids, 2 mM glutamine, 100 units of penicillin/streptomycin, 100 mM b-mercaptoethanol, and 500 units/mL leukemia inhibitory factor (LIF).

The V_H_1-2^JH2^/Vκ3-20^hTdT^-rearranging mouse was generated by blastocyst injection of the ES cells described above and several rounds of breeding to get germline transmission and homozygous mice. The V_H_1-2^JH2^/Vκ1-33/Vκ3-20^hTdT^-rearranging mouse was generated by cross-breeding of V_H_1-2^JH2^/Vκ3-20^hTdT^ and V_H_1-2^JH2^/Vκ1-33 mice. Thus, human Vκ1-33 and Vκ3-20 segments were used on separated alleles. All mouse experiments were performed under protocol 20-08-4242R approved by the Institutional Animal Care and Use Committee of Boston Children’s Hospital.

### Immunogen and Immunization.

Immunogen eOD-GT8 60mer was made as previously described (49). For immunization, each 8 to 12-wk-old mouse was immunized with 200 μL mixture that contain 25 μg filter-sterilized immunogen and 60 μg of poly I:C in PBS by intraperitoneal injection.

### Splenic B Cell, GC B Cell Purification and Antigen-Specific GC B Cell Sorting.

Splenic B cells used for HTGTS-Rep-seq were purified from unimmunized 5 to 8-wk-old mice by MACS® Microbeads according to the manufacturer’s protocol. In brief, spleens were dissected out from unimmunized mice, prepared into single-cell suspensions, and stained with anti-B220 Microbeads for 20 min at 4 °C. The splenic B cells were collected using the LS column and MACS™ Separator. GC B cells used for Rep-SHM-seq were purified from 8 to 12-wk-old mice after eOD-GT8 60mer immunization. GC B cells were sorted for the phenotype B220^+^ (BV711: BioLegend 103255), CD95^+^ (PE-Cy7: eBioscience 557653) and GL7^+^ (PE: BioLegend 144607). CD4-binding site-specific GC B cells for single-cell RT-PCR were further selected for the phenotype eOD-GT8 Fc^+^ and ∆eOD-GT8 Fc^−^. The eOD-GT8 Fc was conjugated with Alexa Fluor 647 fluorescence (Thermo Fisher Scientific A30009). The ∆eOD-GT8 Fc was conjugated with Biotin (Thermo Fisher Scientific A30010) and then stained with SA-BV605 (BioLegend 405229).

### Human Tonsil Mature Naive B Cell Isolation and Genomic DNA Extraction.

Human tonsils were obtained from discarded tissues as part of a routine tonsillectomy from patients at Boston Children’s Hospital. Human tissues were obtained under the IRB approved protocol IRB-P00026526, to J.P.M. Tonsils were minced in RPMI 1640 with 10% FBS and forced through a 45 μm mesh and washed twice with media. The single-cell suspension was stained with 7-AAD (Biolegend) for viability and antibodies directed against human CD19 (APC clone SJ25-C1, Thermo Fisher Scientific), CD38 (PE-Cy7 clone HB-7, Biolegend), IgD (FITC polyclonal, Thermo fisher) and CD27 (APC-Cy7 clone M-T271, Biolegend). Live Naive B cells were obtained by sorting the stained cells using a FACS Aria (BD Biosciences) as 7-AAD^−^CD19^+^CD38^−^IgD^+^CD27^−^. Genomic DNA from sorted cells was prepared using a DNeasy Blood and Tissue Kit (Qiagen) according to the manufacturer’s protocol.

### HTGTS-Rep-seq and Rep-SHM-seq Analysis.

Ten micrograms of DNA from purified splenic B cells was used for generating HTGTS-Rep-seq libraries as previously described (62). Four bait primers that target mouse Jκ1, Jκ2, Jκ4, and Jκ5 were mixed to capture all Igκ LC repertoire in one library. One bait primer that targets human J_H_2 was used to capture HC repertoire. The sequences of human J_H_2 and mouse Jκ primers were as same as the previously reported (54, 68). These HTGTS-Rep-seq libraries were sequenced by Illumina NextSeq 2 × 150-bp paired end kit analyzed with the HTGTS-Rep-seq pipeline (62). DNA from GC B cells was used for generating Rep-SHM-seq libraries as previously described (68). To capture the full-length V(D)J sequence especially the CDR1 region for intrinsic SHM analysis, we designed bait primers that target human V intron regions. The primer sequences are in *SI Appendix*, Table S3. These Rep-SHM-seq libraries were sequenced by Illumina MiSeq 2 × 300-bp paired end kit analyzed with the Rep-SHM-seq pipeline, which uses IgBLAST to annotate V, D, J, and CDRs for each read (68).The HTGTS-Rep-seq and Rep-SHM-seq data are available through the Gene Expression Omnibus (GEO) database (GSE214884).

### Single-Cell RT-PCR and Monoclonal Antibody Production.

Single-cell RT-PCR was performed as described previously (57). In brief, single antigen-specific GC B cells were sorted into 96-well plate that contain 5 µL lysis buffer in each well. After sorting, we used a primer mixture that specifically targets Cµ, Cγ1, Cγ2a, and Cκ to perform reverse transcription and then two rounds of nested PCR to amplify the V(D)J sequences of V_H_1-2 HC, human Vκ3-20/Vκ1-33 LC, and mouse LC. The first round of PCR was performed at 94 °C for 5 min followed by 25 cycles of 94 °C for 30 s, 60 °C (V_H_1-2) or 58 °C (Vκ3-20/Vκ1-33) for 30 s, 72 °C for 60 s, and final incubation at 72 °C for 5 min. The second round of PCR was performed with 2 µL of unpurified first-round PCR product at 94 °C for 5 min followed by 35 cycles of 94 °C for 30 s, 60 °C (V_H_1-2) or 58 °C (Vκ3-20/Vκ1-33) for 30 s, 72 °C for 60 s, and final incubation at 72 °C for 5 min. The mouse LCs were amplified with different annealing temperatures, 50 °C in the first round of PCR and 45 °C in the second round of PCR, and different amplification cycles, 50 cycles for both PCRs. PCR products were run on agarose gels and sanger sequencing was used to confirm their identity. The V(D)J sequences of these antibodies have been deposited to GenBank (accession Nos. OP598882–OP599353). The primer sequences for V_H_1-2 HC, Vκ3-20 and Vκ1-33 LC amplification are listed in *SI Appendix*, Table S3. The primer sequences for mouse LC amplification were as same as previously reported (79). Genes encoding the antibody Fv regions were synthesized by GenScript and cloned into antibody expression vectors pCW-CHIg-hG1 and pCW-CLIg-hk (GenBank accessions ON512569 and ON512571). Monoclonal antibodies were generated using the Expi293 expression system (Thermo Fisher Scientific) and purified by rProtein A Sepharose Fast Flow resin (Cytiva).

### Carterra Human IgG Capture.

Kinetics and affinity of antibody–antigen interactions were measured on Carterra LSA using HC30M or CMDP Sensor Chip (Carterra) and 1x HBS-EP+ pH 7.4 running buffer (20× stock from Teknova, Cat. No H8022) supplemented with Bovine Serum Albumin (BSA) at 1 mg/mL. Chip surfaces were prepared for ligand capture following Carterra software instructions. In a typical experiment about 1,000 to 1,700 RU of capture antibody (SouthernBiotech Cat no 2047-01) in 10 mM sodium acetate pH 4.5 was amine coupled. Phosphoric Acid 1.7% was our regeneration solution with 30 s contact time and injected three times per each cycle. Solution concentration of ligands was above 10 ug/mL, and the contact time was 10 min as per Carterra manual. Raw sensograms were analyzed using Kinetics software (Carterra), interspot and blank double referencing, Langmuir model. Analyte concentrations were quantified on NanoDrop 2000c Spectrophotometer using absorption signal at 280 nm.

### Analyses of CDR3 Diversity and MH-Mediated V(D)J Recombination.

The lengths of insertion and MH for Vκ to Jκ rearrangement were annotated based on HTGTS-Rep-seq results. Insertion nucleotides can be classified into P (palindromic) nucleotides and N (nontemplate) nucleotides. For a read that can be aligned to the 3' end of V segment or 5' end of J segment, the length of P nucleotides was determined by greedy alignment of read sequence outside the V or J end to the reverse complimentary V or J sequence from the end. And the remaining insertion nucleotides were classified as N nucleotides. The length of MH was determined by the length of overlapping read sequence that could be aligned to both V and J (V_end_on_read – J_start_on_read + 1) after greedy alignment to V and J. CDR3 diversity was represented by the percentage of unique CDR3s for a series of downsampled read numbers (e.g., 20, 50, 100, and 200), which could be viewed as rarefaction and estimated by R package "iNEXT." Welch's *t* test was used to compare the percentage of unique CDR3s between groups.

### Statistical Analysis.

Statistical tests with appropriate underlying assumptions on data distribution and variance characteristics were used. The *t* test was used as indicated in the figure legends. Statistical analysis was performed in Prism (v.8, GraphPad Software).

## Supplementary Material

Appendix 01 (PDF)

## Data Availability

All data needed to evaluate the conclusions of the paper are presented in the paper or deposited on the online database. Nucleotide sequences have been deposited to GenBank (accession Nos. OP598882–OP599353). The next-generation sequencing data reported in this paper have been deposited in the Gene Expression Omnibus (GEO) database under the accession number GSE214884. Previously published data were used for this work [GSE197255 (57)].The computational pipeline of Rep-SHM-Seq and the code for statistical analysis tools used in this study are available at https://github.com/Yyx2626/HTGTSrep.

## References

[1] F. W. Alt, Y. Zhang, F. L. Meng, C. Guo, B. Schwer, Mechanisms of programmed DNA lesions and genomic instability in the immune system. Cell **152**, 417–429 (2013). 23374339 10.1016/j.cell.2013.01.007 PMC4382911

[2] A. Rolink, F. Melchers, Molecular and cellular origins of B lymphocyte diversity. Cell **66**, 1081–1094 (1991). 1913803 10.1016/0092-8674(91)90032-t

[3] T. K. Blackwell, F. W. Alt, Mechanism and developmental program of immunoglobulin gene rearrangement in mammals. Annu. Rev. Genet. **23**, 605–636 (1989). 2694945 10.1146/annurev.ge.23.120189.003133

[4] J. P. Allison, L. L. Lanier, Structure, function, and serology of the T-cell antigen receptor complex. Annu. Rev. Immunol. **5**, 503–540 (1987). 2439105 10.1146/annurev.iy.05.040187.002443

[5] D. H. Raulet, The structure, function, and molecular genetics of the gamma/delta T cell receptor. Annu. Rev. Immunol. **7**, 175–207 (1989). 2653369 10.1146/annurev.iy.07.040189.001135

[6] T. W. LeBien, T. F. Tedder, B lymphocytes: How they develop and function. Blood **112**, 1570–1580 (2008). 18725575 10.1182/blood-2008-02-078071 PMC2518873

[7] W. E. Gathings, A. R. Lawton, M. D. Cooper, Immunofluorescent studies of the development of pre-B cells, B lymphocytes and immunoglobulin isotype diversity in humans. Eur. J. Immunol. **7**, 804–810 (1977). 412679 10.1002/eji.1830071112

[8] M. M. Davis, P. J. Bjorkman, T-cell antigen receptor genes and T-cell recognition. Nature **334**, 395–402 (1988). 3043226 10.1038/334395a0

[9] F. J. Bollum, Calf thymus polymerase. J. Biol. Chem. **235**, 2399–2403 (1960). 13802334

[10] F. W. Alt, D. Baltimore, Joining of immunoglobulin heavy chain gene segments: Implications from a chromosome with evidence of three D-JH fusions. Proc. Natl. Acad. Sci. U.S.A. **79**, 4118–4122 (1982). 6287467 10.1073/pnas.79.13.4118 PMC346588

[11] S. Gilfillan, A. Dierich, M. Lemeur, C. Benoist, D. Mathis, Mice lacking TdT: Mature animals with an immature lymphocyte repertoire. Science **261**, 1175–1178 (1993). 8356452 10.1126/science.8356452

[12] T. Komori, A. Okada, V. Stewart, F. W. Alt, Lack of N regions in antigen receptor variable region genes of TdT-deficient lymphocytes. Science **261**, 1171–1175 (1993). 8356451 10.1126/science.8356451

[13] Y. Zhang , The role of short homology repeats and TdT in generation of the invariant gamma delta antigen receptor repertoire in the fetal thymus. Immunity **3**, 439–447 (1995). 7584135 10.1016/1074-7613(95)90173-6

[14] A. J. Feeney, Predominance of the prototypic T15 anti-phosphorylcholine junctional sequence in neonatal pre-B cells. J. Immunol. **147**, 4343–4350 (1991). 1753104

[15] A. J. Feeney, Predominance of VH-D-JH junctions occurring at sites of short sequence homology results in limited junctional diversity in neonatal antibodies. J. Immunol. **149**, 222–229 (1992). 1607655

[16] H. Gu, I. Forster, K. Rajewsky, Sequence homologies, N sequence insertion and JH gene utilization in VHDJH joining: Implications for the joining mechanism and the ontogenetic timing of Ly1 B cell and B-CLL progenitor generation. EMBO J. **9**, 2133–2140 (1990). 2113468 10.1002/j.1460-2075.1990.tb07382.x PMC551934

[17] J. J. Lafaille, A. DeCloux, M. Bonneville, Y. Takagaki, S. Tonegawa, Junctional sequences of T cell receptor gamma delta genes: Implications for gamma delta T cell lineages and for a novel intermediate of V-(D)-J joining. Cell **59**, 859–870 (1989). 2590942 10.1016/0092-8674(89)90609-0

[18] L. K. Aguilar, J. W. Belmont, V gamma 3 T cell receptor rearrangement and expression in the adult thymus. J. Immunol. **146**, 1348–1352 (1991). 1846899

[19] J. F. Elliott, E. P. Rock, P. A. Patten, M. M. Davis, Y. H. Chien, The adult T-cell receptor delta-chain is diverse and distinct from that of fetal thymocytes. Nature **331**, 627–631 (1988). 2963227 10.1038/331627a0

[20] M. Bogue, S. Candeias, C. Benoist, D. Mathis, A special repertoire of alpha: Beta T cells in neonatal mice. EMBO J. **10**, 3647–3654 (1991). 1834457 10.1002/j.1460-2075.1991.tb04931.x PMC453096

[21] A. J. Feeney, Lack of N regions in fetal and neonatal mouse immunoglobulin V-D-J junctional sequences. J. Exp. Med. **172**, 1377–1390 (1990). 1700054 10.1084/jem.172.5.1377 PMC2188672

[22] M. Heller, J. D. Owens, J. F. Mushinski, S. Rudikoff, Amino acids at the site of V kappa-J kappa recombination not encoded by germline sequences. J. Exp. Med. **166**, 637–646 (1987). 3040883 10.1084/jem.166.3.637 PMC2188693

[23] K. D. Victor, K. Vu, A. J. Feeney, Limited junctional diversity in kappa light chains. Junctional sequences from CD43+B220+ early B cell progenitors resemble those from peripheral B cells. J. Immunol. **152**, 3467–3475 (1994). 7511648

[24] T. H. Thai, J. F. Kearney, Distinct and opposite activities of human terminal deoxynucleotidyltransferase splice variants. J. Immunol. **173**, 4009–4019 (2004). 15356150 10.4049/jimmunol.173.6.4009

[25] Y. S. Li, K. Hayakawa, R. R. Hardy, The regulated expression of B lineage associated genes during B cell differentiation in bone marrow and fetal liver. J. Exp. Med. **178**, 951–960 (1993). 8350062 10.1084/jem.178.3.951 PMC2191150

[26] T. H. Thai, M. M. Purugganan, D. B. Roth, J. F. Kearney, Distinct and opposite diversifying activities of terminal transferase splice variants. Nat. Immunol. **3**, 457–462 (2002). 11938351 10.1038/ni788

[27] C. L. Benedict, J. F. Kearney, Increased junctional diversity in fetal B cells results in a loss of protective anti-phosphorylcholine antibodies in adult mice. Immunity **10**, 607–617 (1999). 10367906 10.1016/s1074-7613(00)80060-6

[28] A. Aono , Forced expression of terminal deoxynucleotidyl transferase in fetal thymus resulted in a decrease in gammadelta T cells and random dissemination of Vgamma3Vdelta1 T cells in skin of newborn but not adult mice. Immunology **99**, 489–497 (2000). 10792495 10.1046/j.1365-2567.2000.00987.x PMC2327197

[29] X. Wu , Rational design of envelope identifies broadly neutralizing human monoclonal antibodies to HIV-1. Science **329**, 856–861 (2010). 20616233 10.1126/science.1187659 PMC2965066

[30] J. F. Scheid , Sequence and structural convergence of broad and potent HIV antibodies that mimic CD4 binding. Science **333**, 1633–1637 (2011). 21764753 10.1126/science.1207227 PMC3351836

[31] X. Wu , Focused evolution of HIV-1 neutralizing antibodies revealed by structures and deep sequencing. Science **333**, 1593–1602 (2011). 21835983 10.1126/science.1207532 PMC3516815

[32] T. Zhou , Multidonor analysis reveals structural elements, genetic determinants, and maturation pathway for HIV-1 neutralization by VRC01-class antibodies. Immunity **39**, 245–258 (2013). 23911655 10.1016/j.immuni.2013.04.012 PMC3985390

[33] I. S. Georgiev , Delineating antibody recognition in polyclonal sera from patterns of HIV-1 isolate neutralization. Science **340**, 751–756 (2013). 23661761 10.1126/science.1233989

[34] T. Zhou , Structural repertoire of HIV-1-neutralizing antibodies targeting the CD4 supersite in 14 donors. Cell **161**, 1280–1292 (2015). 26004070 10.1016/j.cell.2015.05.007 PMC4683157

[35] X. Wu , Maturation and diversity of the VRC01-antibody lineage over 15 years of chronic HIV-1 infection. Cell **161**, 470–485 (2015). 25865483 10.1016/j.cell.2015.03.004 PMC4706178

[36] J. Huang , Identification of a CD4-binding-site antibody to HIV that evolved near-pan neutralization breadth. Immunity **45**, 1108–1121 (2016). 27851912 10.1016/j.immuni.2016.10.027 PMC5770152

[37] J. Umotoy , Rapid and focused maturation of a VRC01-class HIV broadly neutralizing antibody lineage involves both binding and accommodation of the N276-Glycan. Immunity **51**, 141–154.e6 (2019). 31315032 10.1016/j.immuni.2019.06.004 PMC6642152

[38] S. Hoot , Recombinant HIV envelope proteins fail to engage germline versions of anti-CD4bs bNAbs. PLoS Pathog. **9**, e1003106 (2013). 23300456 10.1371/journal.ppat.1003106 PMC3536657

[39] J. Jardine , Rational HIV immunogen design to target specific germline B cell receptors. Science **340**, 711–716 (2013). 23539181 10.1126/science.1234150 PMC3689846

[40] A. T. McGuire , Engineering HIV envelope protein to activate germline B cell receptors of broadly neutralizing anti-CD4 binding site antibodies. J. Exp. Med. **210**, 655–663 (2013). 23530120 10.1084/jem.20122824 PMC3620356

[41] T. Zhou , Structural basis for broad and potent neutralization of HIV-1 by antibody VRC01. Science **329**, 811–817 (2010). 20616231 10.1126/science.1192819 PMC2981354

[42] J. G. Jardine , Minimally mutated HIV-1 broadly neutralizing antibodies to guide reductionist vaccine design. PLoS Pathog. **12**, e1005815 (2016). 27560183 10.1371/journal.ppat.1005815 PMC4999182

[43] J. G. Jardine , HIV-1 broadly neutralizing antibody precursor B cells revealed by germline-targeting immunogen. Science **351**, 1458–1463 (2016). 27013733 10.1126/science.aad9195 PMC4872700

[44] C. Havenar-Daughton , The human naive B cell repertoire contains distinct subclasses for a germline-targeting HIV-1 vaccine immunogen. Sci. Transl. Med. **10**, eaat0381 (2018). 29973404 10.1126/scitranslmed.aat0381 PMC6145074

[45] P. Dosenovic , Immunization for HIV-1 broadly neutralizing antibodies in human Ig knockin mice. Cell **161**, 1505–1515 (2015). 26091035 10.1016/j.cell.2015.06.003 PMC4604566

[46] J. G. Jardine , HIV-1 VACCINES. Priming a broadly neutralizing antibody response to HIV-1 using a germline-targeting immunogen. Science **349**, 156–161 (2015). 26089355 10.1126/science.aac5894 PMC4669217

[47] A. T. McGuire , Specifically modified Env immunogens activate B-cell precursors of broadly neutralizing HIV-1 antibodies in transgenic mice. Nat. Commun. **7**, 10618 (2016). 26907590 10.1038/ncomms10618 PMC4770077

[48] D. Sok , Priming HIV-1 broadly neutralizing antibody precursors in human Ig loci transgenic mice. Science **353**, 1557–1560 (2016). 27608668 10.1126/science.aah3945 PMC5404394

[49] M. Tian , Induction of HIV neutralizing antibody lineages in mice with diverse precursor repertoires. Cell **166**, 1471–1484.e18 (2016). 27610571 10.1016/j.cell.2016.07.029 PMC5103708

[50] D. Huang , B cells expressing authentic naive human VRC01-class BCRs can be recruited to germinal centers and affinity mature in multiple independent mouse models. Proc. Natl. Acad. Sci. U.S.A. **117**, 22920–22931 (2020). 32873644 10.1073/pnas.2004489117 PMC7502816

[51] X. Wang , Multiplexed CRISPR/CAS9-mediated engineering of pre-clinical mouse models bearing native human B cell receptors. EMBO J. **40**, e105926 (2021). 33258500 10.15252/embj.2020105926 PMC7809789

[52] P. Dosenovic , Anti-HIV-1 B cell responses are dependent on B cell precursor frequency and antigen-binding affinity. Proc. Natl. Acad. Sci. U.S.A. **115**, 4743–4748 (2018). 29666227 10.1073/pnas.1803457115 PMC5939114

[53] R. K. Abbott , Precursor frequency and affinity determine B cell competitive fitness in germinal centers, tested with germline-targeting HIV vaccine immunogens. Immunity **48**, 133–146.e6 (2018). 29287996 10.1016/j.immuni.2017.11.023 PMC5773359

[54] T. Bradley , Immune checkpoint modulation enhances HIV-1 antibody induction. Nat. Commun. **11**, 948 (2020). 32075963 10.1038/s41467-020-14670-w PMC7031230

[55] C. Guo , CTCF-binding elements mediate control of V(D)J recombination. Nature **477**, 424–430 (2011). 21909113 10.1038/nature10495 PMC3342812

[56] S. Jain, Z. Ba, Y. Zhang, H. Q. Dai, F. W. Alt, CTCF-binding elements mediate accessibility of RAG substrates during chromatin scanning. Cell **174**, 102–116. e14 (2018). 29804837 10.1016/j.cell.2018.04.035 PMC6026039

[57] S. Luo , An antibody from single human VH-rearranging mouse neutralizes All SARS-CoV-2 variants through BA.5 by inhibiting membrane fusion. Sci. Immunol. **7**, eadd5446 (2022), 10.1126/sciimmunol.add5446. 35951767 PMC9407951

[58] K. J. Jackson, M. J. Kidd, Y. Wang, A. M. Collins, The shape of the lymphocyte receptor repertoire: Lessons from the B cell receptor. Front. Immunol. **4**, 263 (2013). 24032032 10.3389/fimmu.2013.00263 PMC3759170

[59] C. Yacoob , Differences in allelic frequency and CDRH3 region limit the engagement of HIV Env immunogens by putative VRC01 neutralizing antibody precursors. Cell Rep. **17**, 1560–1570 (2016). 27806295 10.1016/j.celrep.2016.10.017 PMC5207042

[60] M. Bonsignori , Inference of the HIV-1 VRC01 antibody lineage unmutated common ancestor reveals alternative pathways to overcome a key glycan barrier. Immunity **49**, 1162–1174.e8 (2018). 30552024 10.1016/j.immuni.2018.10.015 PMC6303191

[61] Y. Xiang, S. K. Park, W. T. Garrard, A major deletion in the Vkappa-Jkappa intervening region results in hyperelevated transcription of proximal Vkappa genes and a severely restricted repertoire. J. Immunol. **193**, 3746–3754 (2014). 25187654 10.4049/jimmunol.1401574 PMC4170056

[62] S. G. Lin , Highly sensitive and unbiased approach for elucidating antibody repertoires. Proc. Natl. Acad. Sci. U.S.A. **113**, 7846–7851 (2016). 27354528 10.1073/pnas.1608649113 PMC4948367

[63] H. Q. Dai , Loop extrusion mediates physiological Igh locus contraction for RAG scanning. Nature **590**, 338–343 (2021). 33442057 10.1038/s41586-020-03121-7 PMC9037962

[64] A. P. West Jr., R. Diskin, M. C. Nussenzweig, P. J. Bjorkman, Structural basis for germ-line gene usage of a potent class of antibodies targeting the CD4-binding site of HIV-1 gp120. Proc. Natl. Acad. Sci. U.S.A. **109**, E2083–E2090 (2012). 22745174 10.1073/pnas.1208984109 PMC3409792

[65] B. Briney , Tailored immunogens direct affinity maturation toward HIV neutralizing antibodies. Cell **166**, 1459–1470.e11 (2016). 27610570 10.1016/j.cell.2016.08.005 PMC5018249

[66] Y. R. Lin , HIV-1 VRC01 germline-targeting immunogens select distinct epitope-specific B cell receptors. Immunity **53**, 840–851.e6 (2020). 33053332 10.1016/j.immuni.2020.09.007 PMC7735217

[67] D. F. Robbiani , Convergent antibody responses to SARS-CoV-2 in convalescent individuals. Nature **584**, 437–442 (2020). 32555388 10.1038/s41586-020-2456-9 PMC7442695

[68] H. Chen , BCR selection and affinity maturation in Peyer’s patch germinal centres. Nature **582**, 421–425 (2020). 32499646 10.1038/s41586-020-2262-4 PMC7478071

[69] M. Medina-Ramirez , Design and crystal structure of a native-like HIV-1 envelope trimer that engages multiple broadly neutralizing antibody precursors in vivo. J. Exp. Med. **214**, 2573–2590 (2017). 28847869 10.1084/jem.20161160 PMC5584115

[70] X. Chen , Vaccination induces maturation in a mouse model of diverse unmutated VRC01-class precursors to HIV-neutralizing antibodies with >50% breadth. Immunity **54**, 324–339.e8 (2021). 33453152 10.1016/j.immuni.2020.12.014 PMC8020832

[71] J. P. Allison, W. L. Havran, The immunobiology of T cells with invariant gamma delta antigen receptors. Annu. Rev. Immunol. **9**, 679–705 (1991). 1832874 10.1146/annurev.iy.09.040191.003335

[72] D. H. Raulet , Control of gamma delta T-cell development. Immunol. Rev. **120**, 185–204 (1991). 1650759 10.1111/j.1600-065x.1991.tb00592.x

[73] B. F. Haynes , Strategies for HIV-1 vaccines that induce broadly neutralizing antibodies. Nat. Rev. Immunol. **12**, 1–17 (2022), 10.1038/s41577-022-00753-w. PMC9372928 35962033

[74] T. B. Kepler, Reconstructing a B-cell clonal lineage. I. Statistical inference of unobserved ancestors. F1000Res. **2**, 103 (2013). 24555054 10.12688/f1000research.2-103.v1 PMC3901458

[75] L. Verkoczy , Autoreactivity in an HIV-1 broadly reactive neutralizing antibody variable region heavy chain induces immunologic tolerance. Proc. Natl. Acad. Sci. U.S.A. **107**, 181–186 (2010). 20018688 10.1073/pnas.0912914107 PMC2806760

[76] C. Doyle-Cooper , Immune tolerance negatively regulates B cells in knock-in mice expressing broadly neutralizing HIV antibody 4E10. J. Immunol. **191**, 3186–3191 (2013). 23940276 10.4049/jimmunol.1301285 PMC3773228

[77] Y. Chen , Common tolerance mechanisms, but distinct cross-reactivities associated with gp41 and lipids, limit production of HIV-1 broad neutralizing antibodies 2F5 and 4E10. J. Immunol. **191**, 1260–1275 (2013). 23825311 10.4049/jimmunol.1300770 PMC3725147

[78] T. H. Thai , Regulation of the germinal center response by microRNA-155. Science **316**, 604–608 (2007). 17463289 10.1126/science.1141229

[79] T. Tiller, C. E. Busse, H. Wardemann, Cloning and expression of murine Ig genes from single B cells. J. Immunol. Methods **350**, 183–193 (2009). 19716372 10.1016/j.jim.2009.08.009

