# Supplementary material for: Humanized V(D)J-rearranging and TdT-expressing mouse vaccine models with physiological HIV-1 broadly neutralizing antibody precursors
Source: Proc Natl Acad Sci U S A. 2022 Dec 27;120(1):e2217883120. doi: 10.1073/pnas.2217883120 (PMC9910454; doi:10.1073/pnas.2217883120)
Supplement: Supplementary file 1 — Appendix 01 (PDF) [file pnas.2217883120.sapp.pdf]

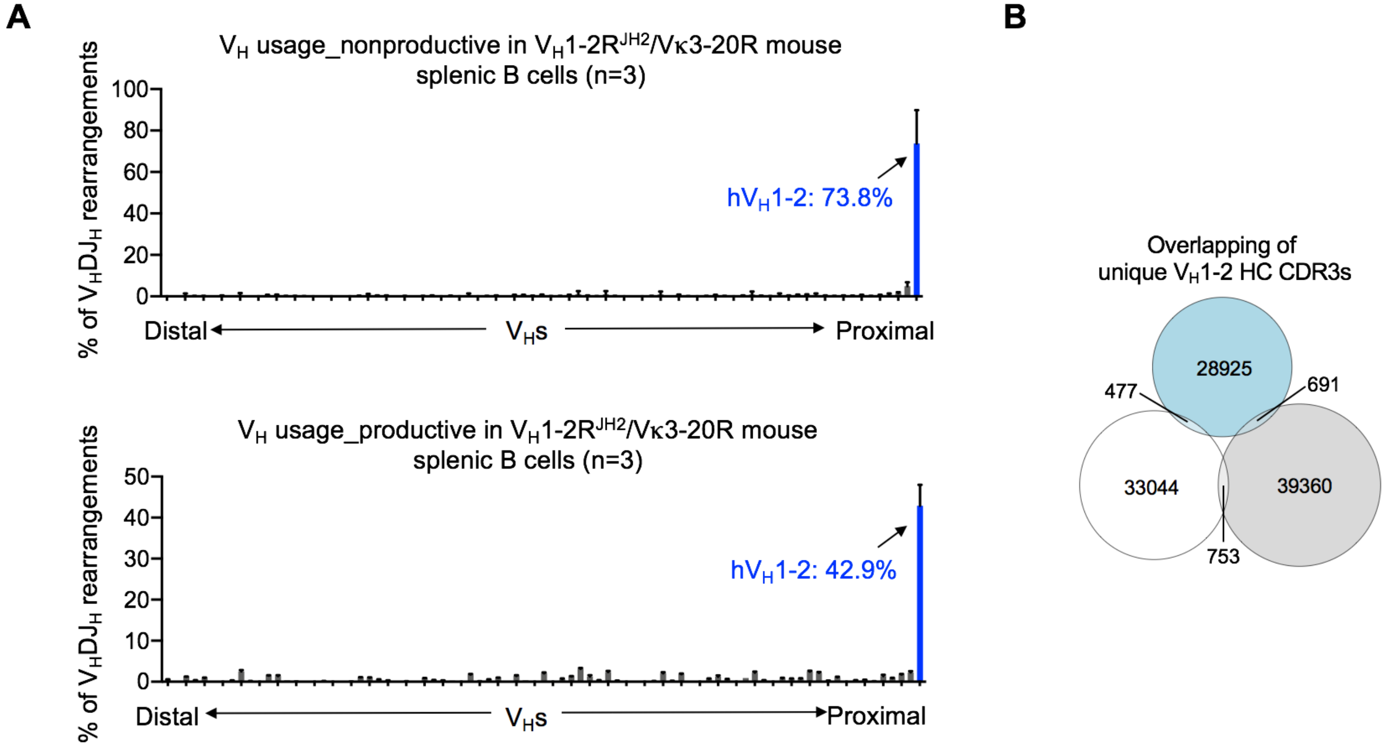

Figure S1. Characterization of V_H1-2^{JH2}-rearranging heavy chain.

(A) HTGTS-rep-seq analyses of V_H non-productive (Upper) and productive (Bottom) rearrangements in V_H1-2^{JH2}/Vκ3-20-rearranging splenic B cells. The histogram displays the percent of nonproductive or productive rearrangements of each V_H among all V_HDJ_H nonproductive or productive rearrangements. The frequencies of V_H nonproductive rearrangements represent the V_H usages in primary V(D)J rearrangements, as the nonproductive allele was not under selection during B cell development. Data were average of 3 experimental repeats with error bars representing SDs.

(B) Venn diagram showed the V_H1-2 HC CDR3 diversity. The unique reads derived from the same libraries in Fig. 1B.

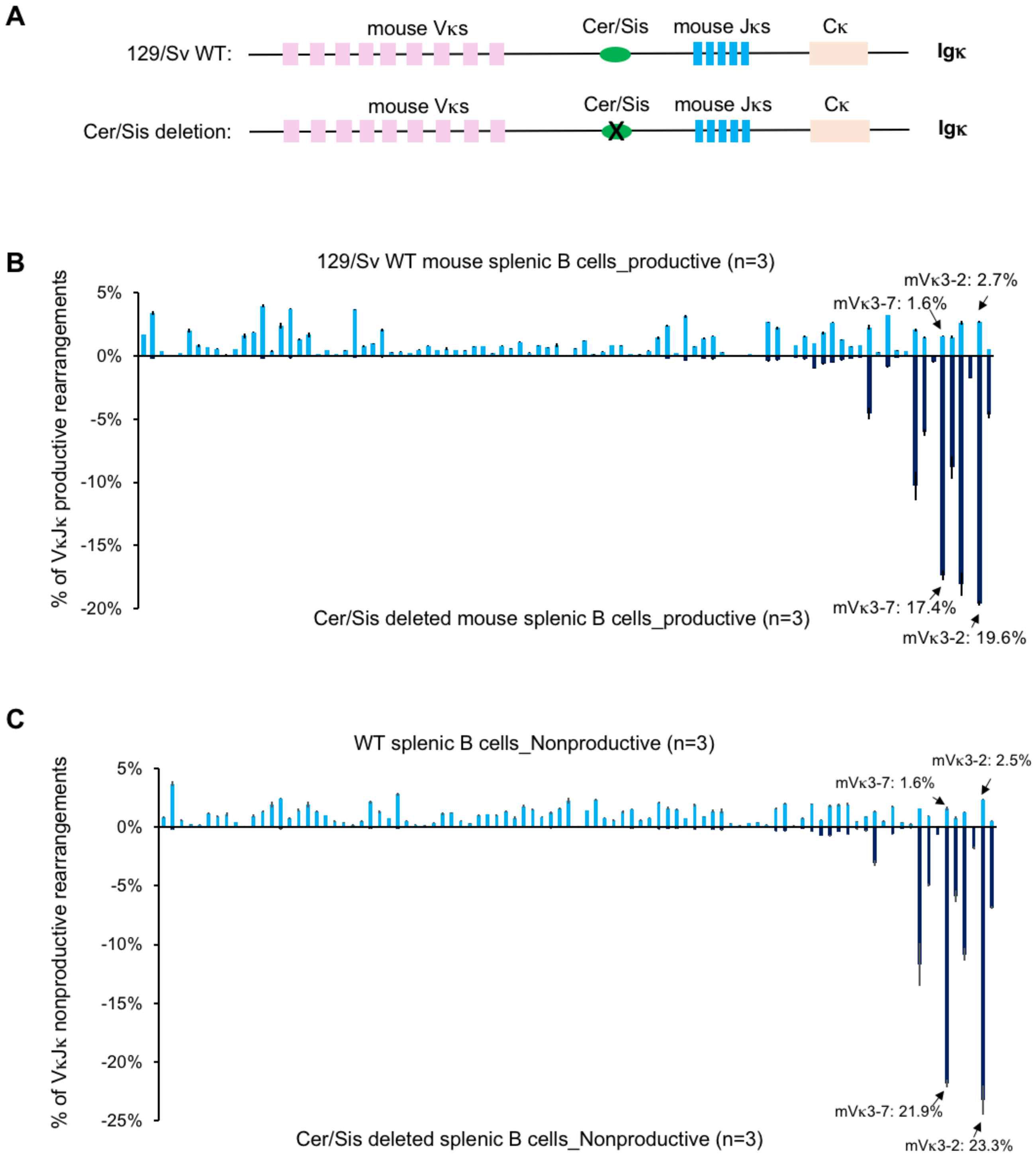
A
129/Sv WT:
mouse Vκs
Cer/Sis
mouse Jκs
Cκ
Igκ
Cer/Sis deletion:
mouse Vκs
Cer/Sis
mouse Jκs
Cκ
Igκ
B
129/Sv WT mouse splenic B cells_productive (n=3)
% of VκJκ productive rearrangements
5%
0%
-5%
-10%
-15%
-20%
mVκ3-2: 2.7%
mVκ3-7: 1.6%
Cer/Sis deleted mouse splenic B cells_productive (n=3)
mVκ3-7: 17.4%
mVκ3-2: 19.6%
C
WT splenic B cells_Nonproductive (n=3)
% of VκJκ nonproductive rearrangements
5%
0%
-5%
-10%
-15%
-20%
-25%
mVκ3-2: 2.5%
mVκ3-7: 1.6%
Cer/Sis deleted splenic B cells_Nonproductive (n=3)
mVκ3-7: 21.9%
mVκ3-2: 23.3%

Figure S2. Cer/sis deletion in wild-type mice increased the utilizations of proximal Vκs, including Vκ3-2 and Vκ3-7.

(A) Illustration of Cer/sis deletion in the Igκ locus. The strategy of Cer/sis deletion was the same as recently described (57).

(B) HTGTS-rep-seq analyses of Vκ usages in wild type (Upper) and Cer/Sis deleted (Bottom) mouse splenic B cells. The x axis lists all functional Vκs from the distal to the *Jκ*-proximal ends. The histogram displays the percent usage of each Vκ among all productive VκJκ rearrangements. The productive Vκ rearrangements in splenic B cells represent the Vκ usage in the naïve BCR repertoire. The data in wild type mouse splenic B cells were derived from our recent study (57).

(C) HTGTS-rep-seq analyses of Vκ nonproductive rearrangements in wild type (Upper) and Cer/sis deleted (Bottom) splenic B cells. The histogram displays the percent of nonproductive rearrangements of each Vκ among all nonproductive VκJκ rearrangements. The percentage of Vκ segments in nonproductive rearrangements represents the V usage in primary V(D)J recombination. The data in wild type mouse splenic B cells were derived from our recent study (57).

Data from (B) and (C) were average of 3 experimental repeats with error bars representing SDs.

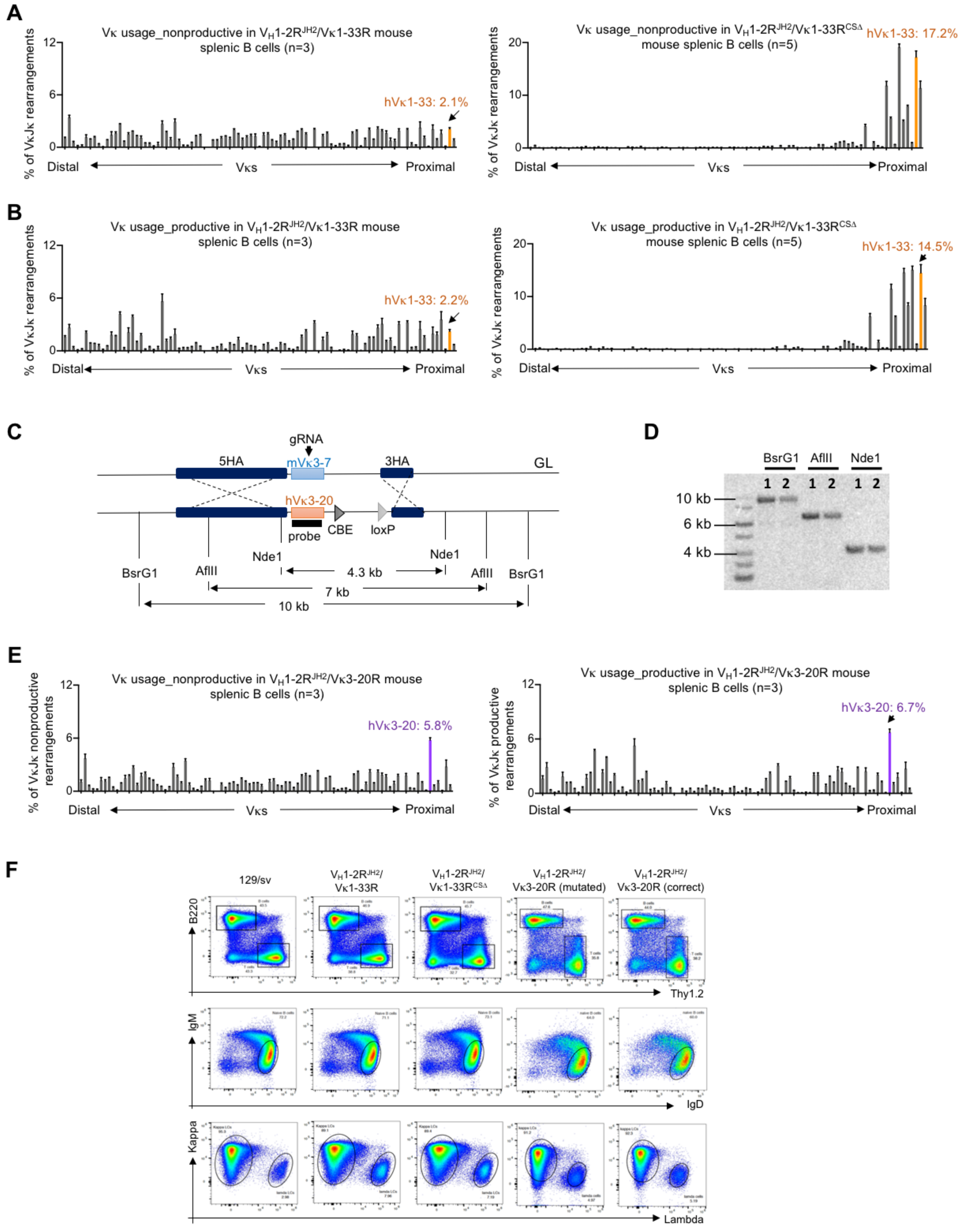

A
Vκ usage_nonproductive in VH1-2RJH2/Vκ1-33R mouse splenic B cells (n=3)
hVκ1-33: 2.1%
% of VκJκ rearrangements
Distal
Vκs
Proximal
Vκ usage_nonproductive in VH1-2RJH2/Vκ1-33RCSΔ mouse splenic B cells (n=5)
hVκ1-33: 17.2%
B
Vκ usage_productive in VH1-2RJH2/Vκ1-33R mouse splenic B cells (n=3)
hVκ1-33: 2.2%
Vκ usage_productive in VH1-2RJH2/Vκ1-33RCSΔ mouse splenic B cells (n=5)
hVκ1-33: 14.5%
C
gRNA
5HA
mVκ3-7
3HA
GL
hVκ3-20
probe
CBE
loxP
Nde1
Nde1
BsrG1
AflII
AflII
BsrG1
4.3 kb
7 kb
10 kb
D
BsrG1
AflII
Nde1
1 2
10 kb
6 kb
4 kb
E
Vκ usage_nonproductive in VH1-2RJH2/Vκ3-20R mouse splenic B cells (n=3)
hVκ3-20: 5.8%
% of VκJκ nonproductive rearrangements
Vκ usage_productive in VH1-2RJH2/Vκ3-20R mouse splenic B cells (n=3)
hVκ3-20: 6.7%
% of VκJκ productive rearrangements
F
129/sv
VH1-2RJH2/ Vκ1-33R
VH1-2RJH2/ Vκ1-33RCSΔ
VH1-2RJH2/ Vκ3-20R (mutated)
VH1-2RJH2/ Vκ3-20R (correct)
B220
Thy1.2
IgM
IgD
Kappa
Lambda

Figure S3. Generation and characterization of the human Vκ-rearranging light chains.

(A) HTGTS-rep-seq analyses of Vκ nonproductive rearrangements in V_H1-2R^{JH2}/Vκ1-33R mouse splenic B cells (Left) and V_H1-2R^{JH2}/Vκ1-33R$^{CS\Delta}$ mouse splenic B cells (Right). The histogram displays the percent of nonproductive rearrangements of each Vκ among all nonproductive VκJκ rearrangements. The Vκ1-33 was labeled in orange. The percentage of Vκ segments in nonproductive rearrangements represents the V usage in primary V(D)J recombination.

(B) HTGTS-rep-seq analyses of Vκ productive rearrangements in V_H1-2R^{JH2}/Vκ1-33R mouse splenic B cells (Left) and V_H1-2R^{JH2}/Vκ1-33R$^{CS\Delta}$ mouse splenic B cells (Right). The histogram displays the percent of productive rearrangements of each Vκ among all productive VκJκ rearrangements. The Vκ1-33 was labeled in orange.

(C) The diagram, not drawn to scale, illustrates the restriction digests and Southern probe that were used to differentiate the region before (GL) and after Vκ3-20 replacement (Vκ3-20-rearranging allele).

(D) Southern analysis of positive ES clones that showed in (C).

(E) HTGTS-rep-seq analyses of Vκ nonproductive (Left) or productive (Right) rearrangements in V_H1-2^{JH2}/Vκ3-20-rearranging splenic B cells. The Vκ3-20 was labeled in purple.

(F) FACS analyses of splenic B cells from wild-type 129/Sv, V_H1-2R^{JH2}/Vκ1-33R, V_H1-2R^{JH2}/Vκ1-33R$^{CS\Delta}$, V_H1-2R^{JH2}/Vκ3-20R (mutated) and V_H1-2R^{JH2}/Vκ3-20R (correct) mice. We repeated these analyses in 3 mice and they show similar results.

Data from (A), (B) and (E) were average of >=3 experimental repeats with error bars representing SDs.

1~100nt

Vκ3-20 DNA sequence (correct): GAAATTGTGTTGACGCAGTCTCCAGGCACCCTGTCTTTGTCTCCAGGGGAAAGAGCCACCCTCTCCTGCAGGGCCAGTCAGAGTGTTAGCAGCAGCTACT
Vκ3-20 DNA sequence with a point mutation: GAAATTGTGTTGACGCAGTCTCCAGGCACCCTGTCTTTGTCTCCAGGGGAAAGAGCCACCCTCTCCTGCAGGGCCAGTCAGA**T**TGTTAGCAGCAGCTACT

101~200nt

Vκ3-20 DNA sequence (correct): TAGCCTGGTACCAGCAGAAACCTGGCCAGGCTCCCAGGCTCCTCATCTATGGTGCATCCAGCAGGGCCACTGGCATCCCAGACAGGTTCAGTGGCAGTGG
Vκ3-20 DNA sequence with a point mutation: TAGCCTGGTACCAGCAGAAACCTGGCCAGGCTCCCAGGCTCCTCATCTATGGTGCATCCAGCAGGGCCACTGGCATCCCAGACAGGTTCAGTGGCAGTGG

201~290nt

Vκ3-20 DNA sequence (correct): GTCTGGGACAGACTTCACTCTCACCATCAGCAGACTGGAGCCTGAAGATTTTGCAGTGTATTACTGTCAGCAGTATGGTAGCTCACCTCC
Vκ3-20 DNA sequence with a point mutation: GTCTGGGACAGACTTCACTCTCACCATCAGCAGACTGGAGCCTGAAGATTTTGCAGTGTATTACTGTCAGCAGTATGGTAGCTCACCTCC

Vκ3-20 amino acid sequence (correct): EIVLTQSPGTLSLSPGERATLSCRASQSVSSSYLAWYQQKPGQAPRLLIYGASSRATGIPDRFSGSGSGTDFTLTISRLEPEDFAVYYCQQYGSSP
Vκ3-20 amino acid sequence with a mutation: EIVLTQSPGTLSLSPGERATLSCRASQ**I**VSSSYLAWYQQKPGQAPRLLIYGASSRATGIPDRFSGSGSGTDFTLTISRLEPEDFAVYYCQQYGSSP

Figure S4. Mutation correction on the Vκ3-20 allele.
A point mutation labeled in red on the nucleotide (upper) and amino acid (bottom) sequences of Vκ3-20 LC.

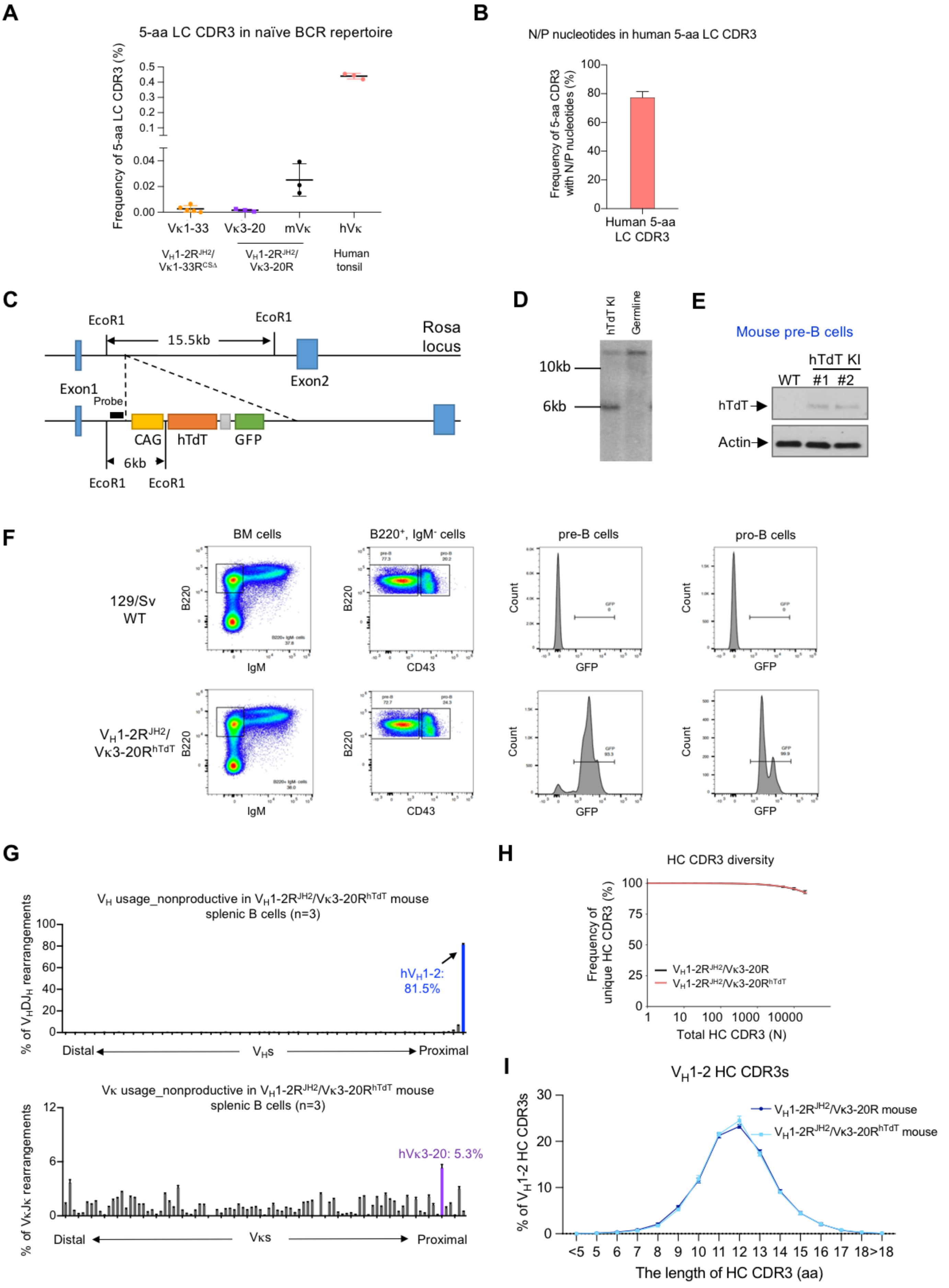
A
5-aa LC CDR3 in naïve BCR repertoire
Frequency of 5-aa LC CDR3 (%)
0.5
0.4
0.3
0.2
0.1
0.04
0.02
0.00
Vκ1-33
Vκ3-20
mVκ
hVκ
VH1-2RJH2/ Vκ1-33RCSΔ
VH1-2RJH2/ Vκ3-20R
Human tonsil
B
N/P nucleotides in human 5-aa LC CDR3
Frequency of 5-aa CDR3 with N/P nucleotides (%)
100
80
60
40
20
0
Human 5-aa LC CDR3
C
EcoR1
15.5kb
EcoR1
Rosa locus
Exon1
Exon2
Probe
CAG
hTdT
GFP
6kb
EcoR1
EcoR1
D
hTdT KI
Germline
10kb
6kb
E
Mouse pre-B cells
hTdT KI
WT
#1
#2
hTdT
Actin
F
BM cells
B220+, IgM- cells
pre-B cells
pro-B cells
129/Sv WT
VH1-2RJH2/ Vκ3-20RhTdT
B220
IgM
CD43
Count
GFP
G
VH usage_nonproductive in VH1-2RJH2/Vκ3-20RhTdT mouse splenic B cells (n=3)
% of VHDJH rearrangements
100
80
60
40
20
0
hVH1-2: 81.5%
Distal
VHs
Proximal
Vκ usage_nonproductive in VH1-2RJH2/Vκ3-20RhTdT mouse splenic B cells (n=3)
% of VκJκ rearrangements
12
6
0
hVκ3-20: 5.3%
Distal
Vκs
Proximal
H
HC CDR3 diversity
Frequency of unique HC CDR3 (%)
100
75
50
25
0
VH1-2RJH2/Vκ3-20R
VH1-2RJH2/Vκ3-20RhTdT
1
10
100
1000
10000
Total HC CDR3 (N)
I
VH1-2 HC CDR3s
% of VH1-2 HC CDR3s
30
20
10
0
VH1-2RJH2/Vκ3-20R mouse
VH1-2RJH2/Vκ3-20RhTdT mouse
<5
5
6
7
8
9
10
11
12
13
14
15
16
17
18
>18
The length of HC CDR3 (aa)

Figure S5. Enforced human TdT expression in the V_H1-2^{JH2}/Vκ3-20-rearranging mouse models.

(A) Frequency of Vκ3-20, Vκ1-33, mouse Igκ and human Igκ LCs with 5-aa CDR3s in our VRC01-rearranging mouse splenic B cells and human tonsil naïve B cells.

(B) Distribution of N or P nucleotides in human naïve Igκ LCs with 5-aa CDR3s.

(C) The diagram illustrates the restriction digest and southern probe that were used to differentiate the region before and after human TdT knock-in.

(D) Southern analysis of ES clone with hTdT knock-in.

(E) Western Blot of TdT expression in mouse pre-B cells before and after hTdT knock-in. The TdT antibody can detect both human and mouse TdT.

(F) FACS analyses of bone marrow B cells from 129/Sv wild-type and V_H1-2R^{JH2}/Vκ3-20R^{hTdT} mice. The pre-B cells were defined by B220$^+$, IgM$^-$ and CD43$^-$. The pro-B cells were defined by B220$^+$, IgM$^-$ and CD43$^+$. The GFP expression was linked with TdT expression as they shared a same promoter.

(G) HTGTS-rep-seq analyses of nonproductive V_H (upper panel) or Vκ (bottom panel) usages in V_H1-2R^{JH2}/Vκ3-20R^{hTdT} mouse splenic B cells. The x axis represented V_H or Vκ locus from the distal to the *J*-proximal ends. The histogram displays the percent of usage of each V_H or Vκ among all nonproductive V_H(D)J_H or VκJκ rearrangements. The usage of human V_H1-2 was labeled in blue and the usage of human Vκ3-20 was labeled in purple.

(H) The diversity of HC CDR3s in V_H1-2R^{JH2}/Vκ3-20R mouse and V_H1-2R^{JH2}/Vκ3-20R^{hTdT} mouse splenic B cells. The x axis represents the total HC CDR3 number (N). The y axis represents the frequency of unique HC CDR3s among total HC CDR3s. The differences of CDR3 diversities between V_H1-2R^{JH2}/Vκ3-20R mouse and V_H1-2R^{JH2}/Vκ3-20R^{hTdT} are not significant.

(I) Length distribution of HC CDR3s in V_H1-2R^{JH2}/Vκ3-20R and V_H1-2R^{JH2}/Vκ3-20R^{hTdT} mouse splenic B cells. The differences measured by t-test were not significant.

Data from (A), (B), (G) and (I) were mean ± SD of >=3 libraries from different mice.

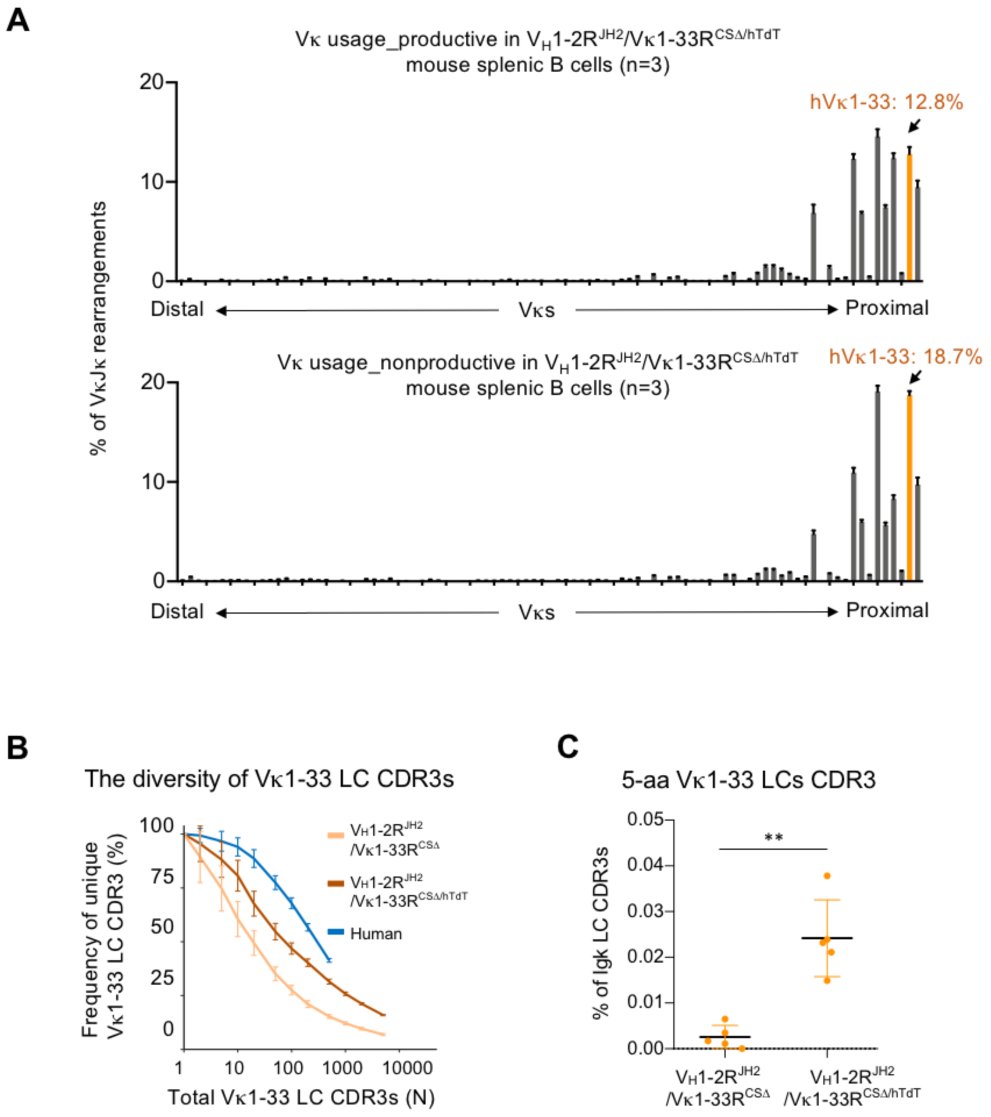

Figure S6. Enforced human TdT expression in the V_H1-2^{JH2}/Vκ1-33R$^{CS\Delta}$-rearranging mouse models.

(A) HTGTS-rep-seq analysis of Vκ productive (Upper) or nonproductive (Bottom) rearrangements in V_H1-2^{JH2}/Vκ1-33$^{CS\Delta/hTdT}$-rearranging splenic B cells. The Vκ1-33 was labeled in orange. The percentage of Vκ segments in nonproductive rearrangements represents the V usage in primary V(D)J recombination.

(B) The diversity of Vκ1-33 LC CDR3s in human, V_H1-2R^{JH2}/Vκ1-33R$^{CS\Delta}$ and V_H1-2R^{JH2}/Vκ1-33R$^{CS\Delta/hTdT}$ mouse naïve B cells. The differences of CDR3 diversities between V_H1-2R^{JH2}/Vκ1-33R$^{CS\Delta}$ and V_H1-2R^{JH2}/Vκ1-33R$^{CS\Delta/hTdT}$ mice are significant when the total CDR3 number is above 10 (p<0.001 for N>=10).

(C) The frequency of 5-aa Vκ1-33 LC CDR3s in V_H1-2R^{JH2}/Vκ1-33R$^{CS\Delta}$ and V_H1-2R^{JH2}/Vκ1-33R$^{CS\Delta/hTdT}$ mouse naïve B cells.

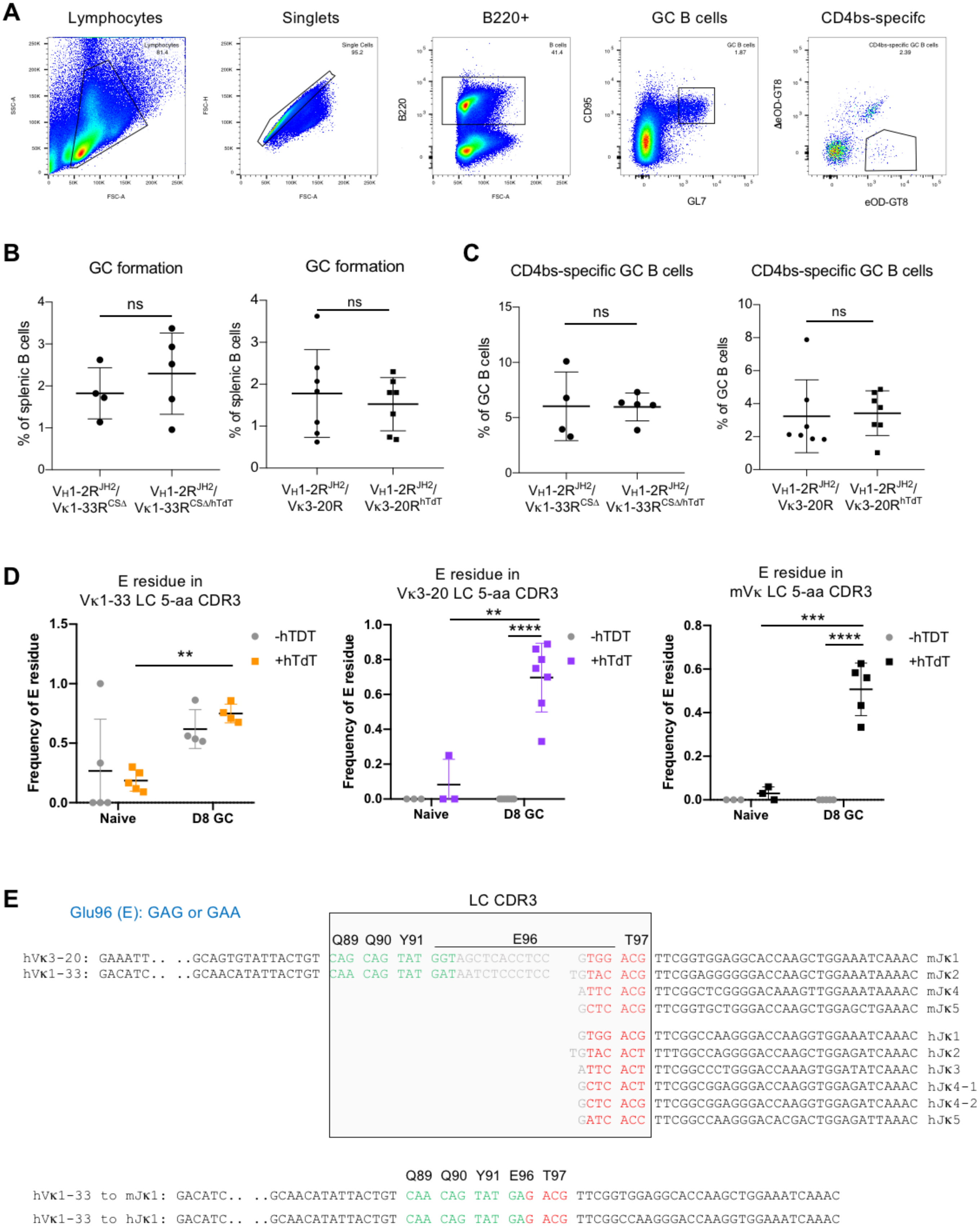
A
Lymphocytes
Singlets
B220+
GC B cells
CD4bs-specifc
SSC-A
FSC-A
FSC-H
B220
CD95
GL7
ΔeOD-GT8
eOD-GT8
B
GC formation
% of splenic B cells
ns
VH1-2R^JH2/ Vκ1-33R^CSΔ
VH1-2R^JH2/ Vκ1-33R^CSΔ/hTdT
VH1-2R^JH2/ Vκ3-20R
VH1-2R^JH2/ Vκ3-20R^hTdT
C
CD4bs-specific GC B cells
% of GC B cells
ns
D
E residue in Vκ1-33 LC 5-aa CDR3
E residue in Vκ3-20 LC 5-aa CDR3
E residue in mVκ LC 5-aa CDR3
Frequency of E residue
-hTDT
+hTdT
Naive
D8 GC
**
****
***
E
Glu96 (E): GAG or GAA
LC CDR3
Q89 Q90 Y91 E96 T97
hVκ3-20: GAAATT.. ..GCAGTGTATTACTGT CAG CAG TAT GGTAGCTCACCTCC GTGG ACG TTCGGTGGAGGCACCAAGCTGGAAATCAAAC mJκ1
hVκ1-33: GACATC.. ..GCAACATATTACTGT CAA CAG TAT GATAATCTCCCTCC TGTAC ACG TTCGGAGGGGGGACCAAGCTGGAAATAAAAC mJκ2
ATTC ACG TTCGGCTCGGGGACAAAGTTGGAAATAAAAC mJκ4
GCTC ACG TTCGGTGCTGGGACCAAGCTGGAGCTGAAAC mJκ5
GTGG ACG TTCGGCCAAGGGACCAAGGTGGAAATCAAAC hJκ1
TGTAC ACT TTTGGCCAGGGGACCAAGCTGGAGATCAAAC hJκ2
ATTC ACT TTCGGCCCTGGGACCAAAGTGGATATCAAAC hJκ3
GCTC ACT TTCGGCGGAGGGACCAAGGTGGAGATCAAAC hJκ4-1
GCTC ACG TTCGGCGGAGGGACCAAGGTGGAGATCAAAC hJκ4-2
GATC ACC TTCGGCCAAGGGACACGACTGGAGATTAAAC hJκ5
Q89 Q90 Y91 E96 T97
hVκ1-33 to mJκ1: GACATC.. ..GCAACATATTACTGT CAA CAG TAT GAG ACG TTCGGTGGAGGCACCAAGCTGGAAATCAAAC
hVκ1-33 to hJκ1: GACATC.. ..GCAACATATTACTGT CAA CAG TAT GAG ACG TTCGGCCAAGGGACCAAGGTGGAAATCAAAC

Figure S7. Human TdT enhanced VRC01-class GC responses induced by eOD-GT8 60mer.

(A) Gating strategy for single cell sorting of eOD-GT8-specific germinal center B cells after eOD-GT8 60mer immunization.

(B) Proportion of GC B cells in $V_H1\text{-}2R^{JH2}/V\kappa1\text{-}33R^{CS\Delta}$, $V_H1\text{-}2R^{JH2}/V\kappa3\text{-}20R$, $V_H1\text{-}2R^{JH2}/V\kappa1\text{-}33R^{CS\Delta/hTdT}$ and $V_H1\text{-}2R^{JH2}/V\kappa3\text{-}20R^{hTdT}$ mice. Each point represented one mouse.

(C) Proportion of CD4bs-specific GC B cells in $V_H1\text{-}2R^{JH2}/V\kappa1\text{-}33R^{CS\Delta}$, $V_H1\text{-}2R^{JH2}/V\kappa3\text{-}20R$, $V_H1\text{-}2R^{JH2}/V\kappa1\text{-}33R^{CS\Delta/hTdT}$ and $V_H1\text{-}2R^{JH2}/V\kappa3\text{-}20R^{hTdT}$ mice. Each dot represents one mouse.

(D) The frequency of Glu96 (E) residue in 5-aa CDR3s of Vκ1-33, Vκ3-20 and mouse LCs before (naïve) and after eOD-GT8 60mer immunization (D8 GC). Each dot represents one mouse.

(E) The Glu96 (E) residue formation in 5-aa CDRs of Vκ1-33, Vκ3-20 and mouse LCs. The Glu (E) amino acid is encoded by GAA or GAG. Both Vκ1-33 and Vκ3-20 can provide the G at the first position, but only Vκ1-33 can provide the A at the second position. On the other side, both mouse and human Jκs cannot provide the G and A at first and second positions, but mouse or human Jκ1 can provide G at the third position. Altogether, in the mouse pre-B cell lacking of TdT expression, the Glu96 (E) is formed when Vκ1-33 joins to mouse Jκ1. Other combinations failed to form the E residue. By examination of the mouse Vκ sequences, only Vκ14-111 can form the E residue in 5-aa LC CDR3 without N region added by TdT. But Vκ14-111 was not observed in the GCs induced by eOD-GT8, probably due to the low affinity of V region to eOD-GT8.

Statistical comparisons in (**B**), (**C**) and (**D**) were performed using unpaired, two-tail t-test. *p <0.05, **p <0.01, ***p <0.001, ****p <0.0001

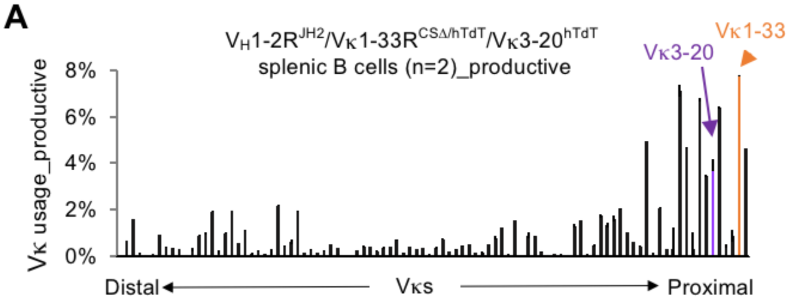

B

Mouse model	V_H1-2R^{JH2}/Vκ1-33R$^{CS\Delta/hTdT}$Vκ3-20^{hTdT}		
Mouse	**#1**	**#2**	**#3**
CD4bs-specific GC B cells	48	48	48
VRC01/Vκ1-33 B cells	18	26	28
VRC01/Vκ3-20 B cells	1	0	0
VRC01/mVκ B cells	0	0	0

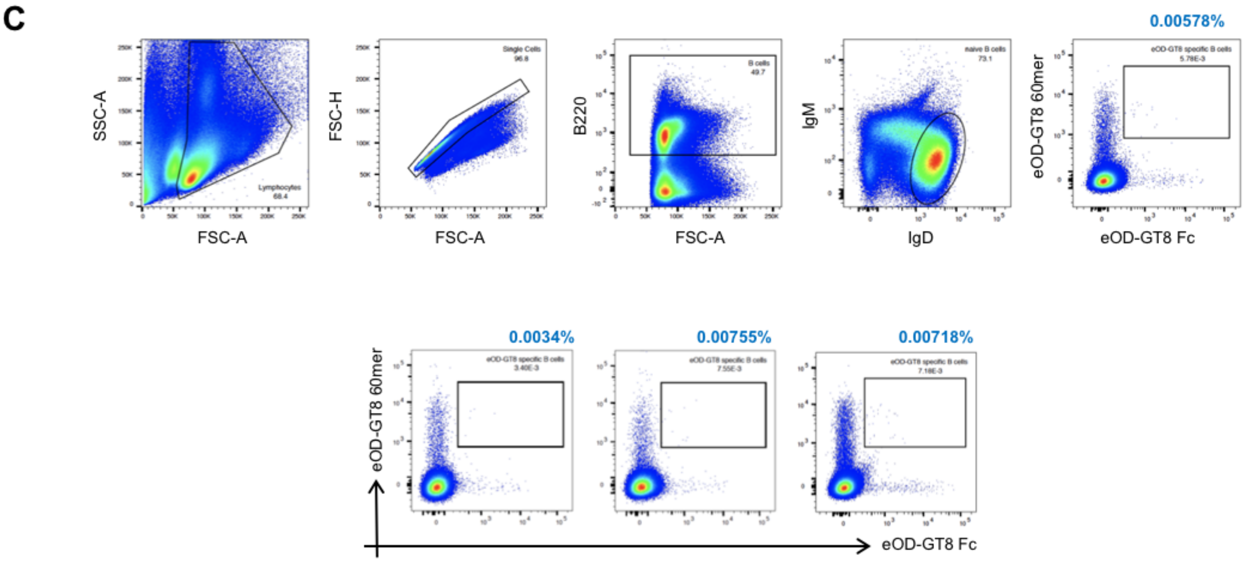

Figure S8. Generation and characterization of V_H1-2^{JH2}/Vκ1-33$^{CS\Delta/hTdT}$/Vκ3-20^{hTdT} and V_H1-2^{JH2}/Vκ1-33/Vκ3-20^{hTdT} -rearranging mouse models.

(A) HTGTS-rep-seq analysis of Vκ usage in V_H1-2R^{JH2}/Vκ1-33R$^{CS\Delta/hTdT}$/Vκ3-20R^{hTdT} mouse splenic B cells. The usage of human Vκ1-33 is labeled in orange, and the usage of human Vκ3-20 is labeled in purple.

(B) Table shown the VRC01-class B cells elicited by eOD-GT8 60mer in V_H1-2R^{JH2}/Vκ1-33R$^{CS\Delta/hTdT}$/Vκ3-20R^{hTdT} mice. 48 CD4bs-specific GC B cells were sorted from each mouse on day 8 GCs post-immunization. The VRC01-class BCRs were identified by single cell RT-PCR following sanger sequencing.

(C) Gating strategy for single cell sorting of eOD-GT8-specific naïve B cells in V_H1-2^{JH2}/Vκ1-33/Vκ3-20^{hTdT}-rearranging mouse models.

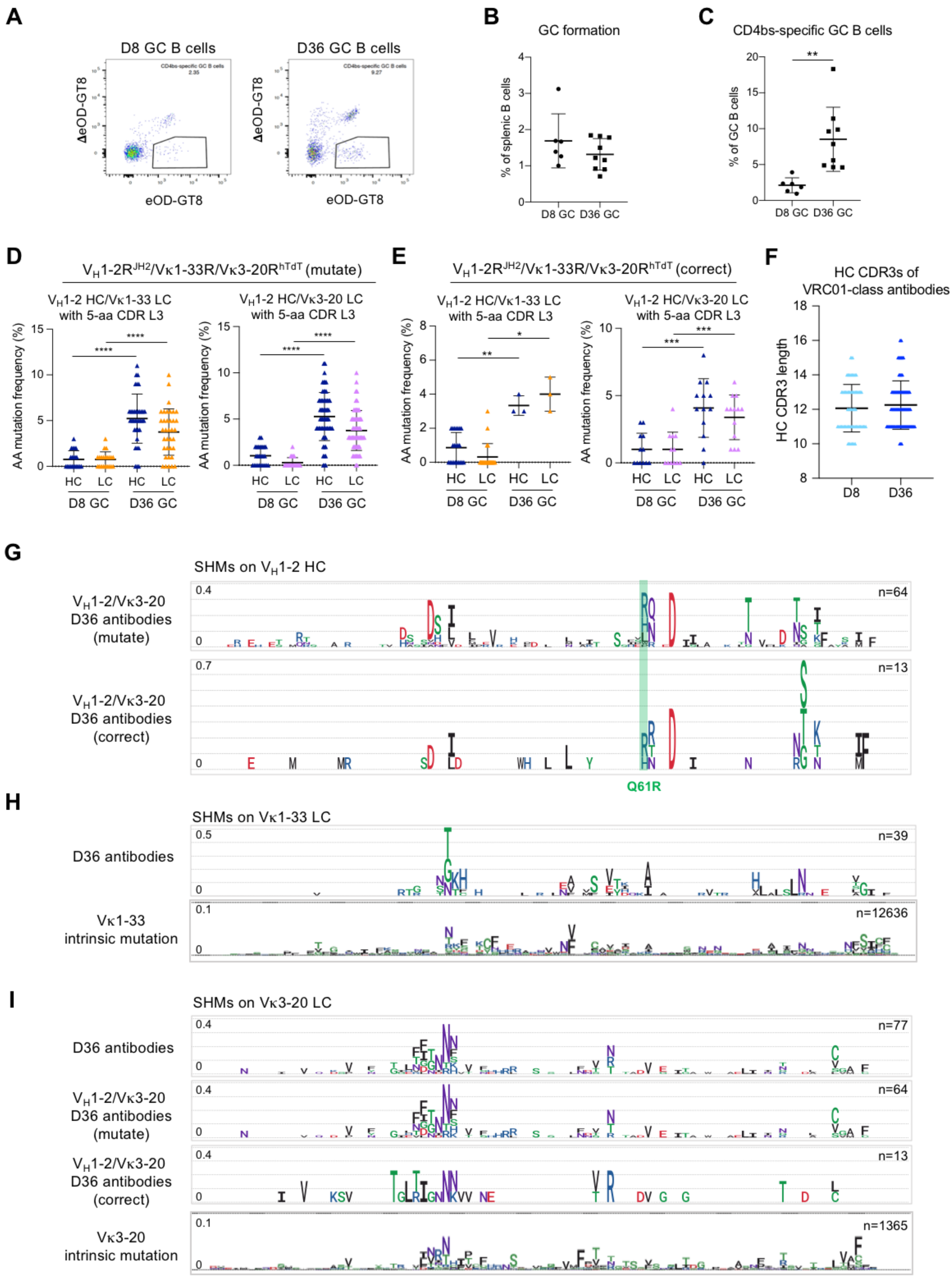
A
D8 GC B cells
D36 GC B cells
ΔeOD-GT8
eOD-GT8
B
GC formation
% of splenic B cells
D8 GC
D36 GC
C
CD4bs-specific GC B cells
% of GC B cells
D
V_H1-2R^{JH2}/Vκ1-33R/Vκ3-20R^{hTdT} (mutate)
V_H1-2 HC/Vκ1-33 LC with 5-aa CDR L3
V_H1-2 HC/Vκ3-20 LC with 5-aa CDR L3
AA mutation frequency (%)
HC
LC
D8 GC
D36 GC
E
V_H1-2R^{JH2}/Vκ1-33R/Vκ3-20R^{hTdT} (correct)
F
HC CDR3s of VRC01-class antibodies
HC CDR3 length
D8
D36
G
SHMs on V_H1-2 HC
V_H1-2/Vκ3-20 D36 antibodies (mutate)
n=64
V_H1-2/Vκ3-20 D36 antibodies (correct)
n=13
Q61R
H
SHMs on Vκ1-33 LC
D36 antibodies
n=39
Vκ1-33 intrinsic mutation
n=12636
I
SHMs on Vκ3-20 LC
D36 antibodies
n=77
V_H1-2/Vκ3-20 D36 antibodies (mutate)
n=64
V_H1-2/Vκ3-20 D36 antibodies (correct)
n=13
Vκ3-20 intrinsic mutation
n=1365

Figure S9. VRC01-class antibodies develop SHM and affinity maturation in GCs induced by eOD-GT8 60mer.

(A) FACS analyses of GC B cells on both day 8 and day 36 post-immunization with eOD-GT8 60mer. The boxed CD4bs-specific GC B cells were sorted for single cell sequencing.

(B) The Proportion of GC B cells in V_H1-2R^{JH2}/Vκ1-33R/Vκ3-20R^{hTdT} mice at day 8 and day 36 post-immunization.

(C) Proportion of CD4bs-specific GC B cells in V_H1-2R^{JH2}/Vκ1-33R/Vκ3-20R^{hTdT} mice at day 8 and day 36 post-immunization.

(D) Amino acid mutation in VRC01-class antibodies cloned from day 8 and day 36 GCs in V_H1-2R^{JH2}/Vκ1-33R/Vκ3-20R^{hTdT} mice with a germline mutation on Vκ3-20 allele. Each dot represents one HC or one LC. The median with interquartile range is plotted.

(E) Amino acid mutation frequency in VRC01-class antibodies cloned from day 8 and day 36 GCs in V_H1-2R^{JH2}/Vκ1-33R/Vκ3-20R^{hTdT} mice with a correct Vκ3-20 allele.

(F) Length distribution of HC CDR3s in all VRC01-class antibodies cloned from day 8 and day 36 GCs.

(G) Mutation frequency of each amino acid on germline-encoded V_H1-2 region of V_H1-2/Vκ3-20 antibodies with (upper) or without (bottom) a germline mutation that cloned from day 36 GCs shown in sequence logo profiles. The distance between dotted horizontal lines representing 0.1 (10%).

(H) Mutation frequency of each amino acid on germline-encoded Vκ1-33 region of VRC01-class antibodies that cloned from day 36 GCs shown in sequence logo profiles. The distance between dotted horizontal lines representing 0.1 (10%). For reference, the intrinsic mutation patterns from non-productive rearrangements are represented below.

(I) Mutation frequency of each amino acid on germline-encoded Vκ3-20 region of V_H1-2/Vκ3-20 antibodies that cloned from day 36 GCs shown in sequence logo profiles. 4 panels from top to bottom showed all V_H1-2/Vκ3-20 antibodies, V_H1-2/Vκ3-20 antibodies with a germline mutation, V_H1-2/Vκ3-20 antibodies with correct sequences, and nonproductive Vκ3-20 sequences that represents the intrinsic mutation pattern. The distance between dotted horizontal lines representing 0.1 (10%).

Statistical comparisons in (C), (D) and (E) were performed using a two-tailed unpaired t test. **p* <0.05, ***p* <0.01, ****p* <0.001, *****p* <0.0001

Table S1. eOD-GT8 binding affinity of VRC01-class antibodies

VRC01-class antibodies	Affinity to eOD-GT8 (KD)
VRC01/VK1-33_D8_5924	1.1E-09
VRC01/VK1-33_D8_7341	1.2E-09
VRC01/VK1-33_D8_5982	5.7E-09
VRC01/VK1-33_D8_5341	1.2E-08
VRC01/VK1-33_D8_5953	1.7E-08
VRC01/VK1-33_D8_7340	1.7E-08
VRC01/VK1-33_D8_5976	3.9E-08
VRC01/VK1-33_D8_5926	5.2E-08
VRC01/VK1-33_D8_5903	5.9E-08
VRC01/VK1-33_D8_5340	1.5E-07
VRC01/VK1-33_D8_5959	2.1E-07
VRC01/VK1-33_D8_5969	4.1E-07
VRC01/VK1-33_D8_5345	4.5E-07
VRC01/VK1-33_D8_5922	3.5E-06
VRC01/VK3-20_D8_5342	2.0E-07
VRC01/VK3-20_D8_7342	1.2E-09
VRC01/VK3-20_D8_7338	1.5E-09
VRC01/VK3-20_D8_5934	4.5E-08
VRC01/VK3-20_D8_7336	1.1E-07
VRC01/VK3-20_D8_5950	1.7E-07
VRC01/VK3-20_D8_5937	3.3E-07
VRC01/VK3-20_D8_5344	6.0E-07
VRC01/VK3-20_D8_5942	6.2E-07
VRC01/VK3-20_D8_5990	8.8E-07
VRC01/VK3-20_D8_5921	1.0E-06
VRC01/VK3-20_D8_5936	1.4E-06
VRC01/VK3-20_D8_5949	no binding
VRC01/mVk_D8_6517	2.3E-09
VRC01/mVk_D8_5927	3.7E-09
VRC01/mVk_D8_5943	3.3E-07
VRC01/mVk_D8_6508	3.7E-07
VRC01/mVk_D8_5952	4.3E-07
VRC01/mVk_D8_5947	4.7E-07
VRC01/VK1-33_D36_6579	6.2E-11
VRC01/VK1-33_D36_6480	9.4E-11
VRC01/VK1-33_D36_6423	9.7E-11
VRC01/VK1-33_D36_6459	1.1E-10
VRC01/VK1-33_D36_6416	2.1E-10
VRC01/VK1-33_D36_6474	1.0E-09
VRC01/VK1-33_D36_6510	1.3E-09
VRC01/VK1-33_D36_6450	4.7E-08
VRC01/VK1-33_D36_6465	1.4E-07
VRC01/VK1-33_D36_5715	5.2E-06
VRC01/VK1-33_D36_6501	no binding
VRC01/VK1-33_D36_6448	no binding
VRC01/VK3-20_D36_6464	8.9E-11
VRC01/VK3-20_D36_6433	1.0E-10
VRC01/VK3-20_D36_6136	2.0E-10
VRC01/VK3-20_D36_6107	2.4E-10
VRC01/VK3-20_D36_6108	3.0E-10
VRC01/VK3-20_D36_6463	3.6E-10
VRC01/VK3-20_D36_6120	4.4E-10
VRC01/VK3-20_D36_6484	1.4E-09
VRC01/VK3-20_D36_6429	9.2E-08
VRC01/VK3-20_D36_6505	9.2E-08
VRC01/VK3-20_D36_6486	1.2E-07
VRC01/VK3-20_D36_6441	7.0E-07
VRC01/VK3-20_D36_6598	4.2E-06
VRC01/VK3-20_D36_6402	no binding
VRC01/VK3-20_D36_6430	no binding
VRC01/VK3-20_D36_6106	no binding
VRC01/mVk_D36_6471	6.7E-10
VRC01/mVk_D36_6455	8.8E-10
VRC01/mVk_D36_6434	1.5E-09
VRC01/mVk_D36_6460	1.7E-08
VRC01/mVk_D36_6466	4.8E-08
VRC01/mVk_D36_6458	1.2E-07

Table S2. VRC01-rearranging mouse models

Epitope	bnAb	Model	Human Ig segment	Other modification	hHC/LC %
CD4 binding site	VRC01	**V_H1-2R^{JH2}/Vκ1-33R^{hTdT}**	hV_H1-2, hJ_H2, hVκ1-33	ΔIGCRI hTdT	hHC(40%), hLC (2%)
		**V_H1-2R^{JH2}/Vκ1-33R$^{CS\Delta/hTdT}$**	hV_H1-2, hJ_H2, hVκ1-33	ΔIGCRI, ΔCer/Sis, hTdT	hHC(40%), hLC (13%)
		**V_H1-2R^{JH2}/Vκ3-20R^{hTdT}**	hV_H1-2, hJ_H2, hVκ3-20	ΔIGCRI, hTdT	hHC(40%), hLC (9%)
		**V_H1-2R^{JH2}/Vκ1-33R$^{CS\Delta/hTdT}$/Vκ3-20R^{hTdT}**	hV_H1-2, hJ_H2, hVκ3-20, hVκ1-33	ΔIGCRI, ΔCer/Sis, hTdT	hHC(40%), hLC (hVκ1-33: 7%; hVκ3-20: 4%)
		**V_H1-2R^{JH2}/Vκ1-33R/Vκ3-20R^{hTdT}**	hV_H1-2, hJ_H2, hVκ3-20, hVκ1-33	ΔIGCRI, hTdT	hHC(40%), hLC (hVκ1-33: 1%; hVκ3-20: 5%)

Table S3. Primer sequences

Method	name	sequence	paper
sgRNA	mVκ3-2 sgRNA1	AGAGAAGCAGGACCCATAGC	Luo et al., 2022
	mVκ3-2 sgRNA2	GTATTTCTGTCAGCAAAGTA	Luo et al., 2022
	mVκ3-7 sgRNA	AGCTAGATGTACTGACACTT	This paper
	Cer/sis deletion-sgRNA1	TCAATACAGCTGCATTAATG	Luo et al., 2022
	Cer/sis deletion-sgRNA2	GAGGAATCTATGTCCTGGAT	Luo et al., 2022
HTGTS primers	mouse Jκ1-Bio	/5BiosG/TTCCCAGCTTTGCTTACGGAG	Chen et al., 2020
	mouse Jκ2-Bio	/5BiosG/ATTCCAACCTCTTGTGGGACAG	Chen et al., 2020
	mouse Jκ4-Bio	/5BiosG/CGCTCAGCTTTCACACTGACTC	Chen et al., 2020
	mouse Jκ5-Bio	/5BiosG/GCCCCTAATCTCACTAGCTTGA	Chen et al., 2020
	mouse Jκ1-red	CAGACATAGACAACGGAAGAAAG	Chen et al., 2020
	mouse Jκ2-red	CAAGGTTAGACTTAGTGAACAAGAG	Chen et al., 2020
	mouse Jκ4-red	CAGAACCAAAACGTCACAAGTAA	Chen et al., 2020
	mouse Jκ5-red	CATGAAAACCTGTGTCTTACACAT	Chen et al., 2020
	human JH2-Bio	/5BiosG/GCTGCAGACCCCAGATACCT	Bradley et al., 2020
	human JH2-Red	TGGACAGAGAAGACTGGGAGG	Bradley et al., 2020
	human Jκ1-Bio	/5BiosG/TGTGCAATCAATTCTCGAGTTTG	This paper
	human Jκ2-Bio	/5BiosG/TCCTCTGTACCTAACCTGGGAAT	This paper
	human Jκ3-Bio	/5BiosG/CCCAATGATTCGTCTATTTGCTC	This paper
	human Jκ4-Bio	/5BiosG/CGCTTGGCTGTTCCTTAAGAT	This paper
	human Jκ5-Bio	/5BiosG/TTGCAACCCATGGCAAATCT	This paper
	human Jκ1-red	ACACAGGGAACAGAAGACACA	This paper
	human Jκ2-red	ATTAGCAACAGTGAAGAATCAGTG	This paper
	human Jκ3-red	GATACAATGGCACTAAAATCTCACG	This paper
	human Jκ4-red	CTCAAACACAAAAACGCTCCAA	This paper
	human Jκ5-red	GTCAATACTGGCCATCAGACC	This paper
	hVH1-2-bio	/5BiosG/TGGACCTGGAGGATCCTCTT	Bradley et al., 2020
	hVH1-2-red	GGGAGATCTCATCCACTTCTGTG	Bradley et al., 2020
	hVκ3-20-bio	/5BiosG/TTCCTCCTGCTACTCTGGCT	This paper
	hVκ3-20-red	CTGGCAACTCTGCTCAGTCAAT	This paper
	hVκ1-33-bio	/5BiosG/ATGGACATGAGGGTCCCTGC	This paper
	hVκ1-33-red	TCCTGCTGCTCTGGCTCTCA	This paper
single Cell RT-PCR	Cmu RT primer	ACC TTC AAG GAT GCT CTT GG	Tian et al., 2016
	Cg1 and Cg2a RT primer	CAG CTG GGA AGG TGT GCA CA	Tian et al., 2016
	Cκ RT primer	GCC TCA CAG GTA TAG CTG TT	Tian et al., 2016
	Cmu outer-R	CCT GGA TGA CTT CAG TGT TG	Tian et al., 2016
	Cg1 and Cg2a outer-R	AGG GAT CCA GAG TTC CAG GT	Tian et al., 2016
	Cκ outer-R	GGA CGC CAT TTT GTC GTT CA	Tian et al., 2016
	Cmu inner-R	AGGGGGAAGACATTTGGGAAGGAC	Tian et al., 2016
	Cg1 inner-R	GCTCAGGGAAATAGCCCTTGAC	Tian et al., 2016
	Cg2a inner-R	ACTCAGGGAAGTAGCCCTTGAC	Tian et al., 2016
	Cκ inner-R	CTTGACATTGATGTCTTTGGGGTAG	Luo et al., 2022
	VH1-2 primer-F	TGG ACC TGG AGG ATC CTC TT	Tian et al., 2016
	Vκ3-20 primer-F	TTC CTC CTG CTA CTC TGG CT	Tian et al., 2016
	Vκ1-33 primer-F	TCAGCTCCTGGGGCTCCTGC	Luo et al., 2022
